# Clinical characteristics and novel mutations of omicron subvariant XBB in Tamil Nadu, India – a cohort study

**DOI:** 10.1016/j.lansea.2023.100272

**Published:** 2023-09-04

**Authors:** Sivaprakasam T. Selvavinayagam, Sree J. Karishma, Kannan Hemashree, Yean K. Yong, Suvaiyarasan Suvaithenamudhan, Manivannan Rajeshkumar, Bijulal Aswathy, Vasudevan Kalaivani, Jayapal Priyanka, Anandhazhvar Kumaresan, Meganathan Kannan, Natarajan Gopalan, Samudi Chandramathi, Ramachandran Vignesh, Amudhan Murugesan, Abdul R. Anshad, Balasubramanian Ganesh, Narcisse Joseph, Hemalatha Babu, Sakthivel Govindaraj, Marie Larsson, Shree L. Kandasamy, Sampath Palani, Kamalendra Singh, Siddappa N. Byrareddy, Vijayakumar Velu, Esaki M. Shankar, Sivadoss Raju

**Affiliations:** a State Public Health Laboratory, Directorate of Public Health and Preventive Medicine, DMS Campus, Teynampet, Chennai, Tamil Nadu 600 006, India; b Infection and Inflammation, Department of Biotechnology, Central University of Tamil Nadu, Thiruvarur 610 005, India; c Laboratory Centre, Xiamen University Malaysia, Sepang, Selangor 43 900, Malaysia; d Department of Bioinformatics, Bishop Heber College, Tiruchirappalli, Tamil Nadu 620 017, India; e Blood and Vascular Biology, Department of Biotechnology, Central University of Tamil Nadu, Thiruvarur 610 005, India; f Department of Epidemiology and Public Health, Central University of Tamil Nadu, Thiruvarur 610 005, India; g Department of Medical Microbiology, University of Malaya, Lembah Pantai, Kuala Lumpur 50603, Malaysia; h Faculty of Medicine, Preclinical Department, Royal College of Medicine Perak, Universiti Kuala Lumpur, Ipoh, Perak 30450, Malaysia; i Department of Microbiology, The Government Theni Medical College and Hospital, Theni, India; j National Institute of Epidemiology, Indian Council of Medical Research, Ayappakkam, Chennai 600 077, India; k Department of Medical Microbiology, Universiti Putra Malaysia, Kuala Lumpur, Malaysia; l Division of Microbiology and Immunology, Department of Pathology and Laboratory Medicine, Emory University School of Medicine, Emory National Primate Research Center, Emory Vaccine Center, Atlanta, GA 30329, USA; m Division of Molecular Medicine and Virology, Department of Biomedical and Clinical Sciences, Linköping University, Sweden; n Bond Life Sciences Center, Department of Veterinary Pathobiology, University of Missouri, Columbia, MO 65211, USA; o Department of Pharmacology and Experimental Neuroscience, University of Nebraska Medical Center, Omaha, NE 68131, USA

**Keywords:** COVID-19, Omicron, XBB variants, Severity, Phylogeny, South India

## Abstract

**Background:**

Despite the continued vaccination efforts, there had been a surge in breakthrough infections, and the emergence of the B.1.1.529 omicron variant of SARS-CoV-2 in India. There is a paucity of information globally on the role of newer XBB variants in community transmission. Here, we investigated the mutational patterns among hospitalised patients infected with the XBB omicron sub-variant, and checked if there was any association between the rise in the number of COVID-19 cases and the observed novel mutations in Tamil Nadu, India.

**Methods:**

Nasopharyngeal and oropharyngeal swabs, collected from symptomatic and asymptomatic COVID-19 patients were subjected to real-time PCR followed by Next Generation Sequencing (NGS) to rule out the ambiguity of mutations in viruses isolated from the patients (n = 98). Using the phylogenetic association, the mutational patterns were used to corroborate clinico-demographic characteristics and disease severity among the patients.

**Findings:**

Overall, we identified 43 mutations in the S gene across 98 sequences, of which two were novel mutations (A27S and T747I) that have not been reported previously with XBB sub-variants in the available literature. Additionally, the XBB sequences from our cohort had more mutations than omicron B.1.1.529. The phylogenetic analysis comprising six major branches clearly showed convergent evolution of XBB. Our data suggests that age, and underlying conditions (e.g., diabetes, hypertension, and cardiovascular disease) or secondary complications confers increased susceptibility to infection rather than vaccination status or prior exposure. Many vaccinated individuals showed evidence of a breakthrough infection, with XBB.3 being the predominant variant identified in the study population.

**Interpretation:**

Our study indicates that the XBB variant is highly evasive from available vaccines and may be more transmissible, and potentially could emerge as the ‘next’ predominant variant, which likely could overwhelm the existing variants of SARS-CoV-2 omicron variants.

**Funding:**

National Health Mission (India), 10.13039/100004441SIDA10.13039/100020422SARC, VINNMER (Sweden), 10.13039/100016958ORIP/10.13039/100000002NIH (USA).

**Research in context**

**Evidence before this study**

Despite being one of the countries with the highest vaccination rates, India witnessed increased number of people getting infected with the different omicron variants. Understanding the mutational landscape of the XBB variant of SARS-CoV-2 is critical to understanding its transmissibility and infection outcomes among different populations. We identified articles from PubMed that included the terms ‘XBB’, ‘immune evasion’, ‘antibody evasion’, and ‘neutralization sensitivity’. Mutational studies regarding the BA.2 sub-variants were identified from India. We found that comprehensive research on mutational epidemiology in a similar context on XBB variants was unavailable from India.

**Added value of this study**

This study comprehensively analysed the different mutational and epidemiological aspects of the emergence of the XBB sub-variant of omicron in southern India. Our study confirms that despite receiving multiple doses of vaccines or prior exposure to SARS-CoV-2 in the pre-omicron era, individuals were not protected from the actively circulating XBB variants during the study period. The severity and morbidity associated with the viral infection were related to an individual's age and underlying conditions.

**Implications of all the available evidence**

Our study indicates that the XBB variant is highly immune evasive; may be more transmissible and might be the ‘next’ predominant variant of SARS-CoV-2 in Tamil Nadu. Continuous surveillance of viral mutations is paramount at the global level as it is critical for the development of an effective vaccine and undertaking of intervention measures against SARS-CoV-2 infection.

## Introduction

SARS-CoV-2 has caused unprecedented socioeconomic losses since its emergence in Wuhan city of China, in early December 2019.^1^, ^2^, ^3^, ^4^, ^5^, ^6^ Over time, the virus accumulated genetic mutations, evolved into several variants that were more pathogenic and/or more infectious or virulent than the parent strain (called the Wuhan-Hu-1 strain) with a surge in transmissibility, and were designated as Variants of Concern (VOCs).^7^ The first VOC B.1.1.7 (alpha variant), identified in the UK in September 2020, was declared a VOC in December 2020. Subsequently, B.1.351 (beta variant), P.1 (gamma variant), and B.1.617.2 (delta variant) were the VOCs that eventually emerged thereafter.^8^ The delta variant was first documented in December 2020 in India. It was held accountable for the emergence of the second wave of the COVID-19 pandemic.^9^ The omicron (B.1.1.529) variant is the latest VOC, which emerged in November 2021.^10^ Since then, several omicron sub-variants have been reported. The omicron variant was characterised by a significantly high (>30) number of mutations in the spike protein (S protein),^10^ compared to previously reported VOCs: alpha (B.1.1.7), beta (B.1.351), gamma (P.1), and delta (B.1.617.2) variants.

Prior studies suggest that the two sub-variants of omicron, the BA.1 and BA.2 can successfully evade antibodies generated during natural infection by the wild-type SARS-CoV-2 and those originating after vaccination.^11^^,^^12^ A similar trend was also seen with the BA.3, BA.4, and BA.5 sub-variants.^13^ Other sub-lineages, namely BA.2.75 and BA.2.75.2, also emerged with greater virulence and transmissibility attributes.^14^ As of October 2022, the World Health Organization (WHO) has placed a new omicron variant, the XBB (a recombinant between BA.2.10.1 and BA.2.75), and its sub-lineages under its surveillance radars. While the increase in disease severity has not been documented thus far, current evidence from Singapore, India, and other countries indicates a higher risk of reinfection with VOCs than the other Omicron sub-lineages.^15^ We investigated if there was any association between the rise in the number of cases and the observed novel mutations in the southern state of Tamil Nadu, India. We also asked if the newly emerging XBB variants were immune evasive and examined if prior infection or vaccination guaranteed protection despite high mutation rates in the viral genome. As a nodal Public Health Agency located in Chennai, State of Tamil Nadu (one among four larger southern states), we set out to document viral evolution, transmission, and COVID-19 severity to investigate possible community transmission. Here, we report the disease dynamics due to the XBB omicron variant as a cause of COVID-19 documented from September 2022 to January 2023.

## Methods

### Study design and participants

The State Public Health Laboratory is a nodal laboratory with whole genome sequencing (WGS) facility, and archives all the COVID-19 samples collected from across the entire state. It also monitors the emergence of new variants of SARS-CoV-2 for developing action plan for disease prevention across Tamil Nadu. In the current study, we collected nasopharyngeal and oropharyngeal swabs from non-hospitalised (n = 175) and hospitalised (n = 69) individuals who had tested positive for COVID-19. COVID-19 diagnosis was made based on standard clinical and laboratory guidelines; the former based on Universal Clinical Criteria 2021 defined by the Centers for Disease Control and Prevention (CDC), Atlanta, USA,^16^ and Ministry of Health and Family Welfare (MoHFW), Government of India, and later confirmed using a commercial TaqPath™ SARS-CoV-2 RT-PCR (Applied Biosystems, ThermoFisher Scientific, Pleasanton, CA) for the qualitative detection of nucleotides or genome sequences of SARS-CoV-2. All samples selected for sequencing had RNA freshly extracted from the primary sample source independent of the material extracted for the initial SARS-CoV-2 RT-PCR assay. RNA extraction was performed using a commercial MagMAX™ Viral/Pathogen II Nucleic Acid isolation kit (Applied Biosystems, Cat. No. A48383, ThermoFisher Scientific, USA) as per the manufacturer's instructions.

### Ethics approval

The Human Ethics Committee (HEC) of the Madras Medical College (MMC) (EC No. 03092021) reviewed and approved the study methodology and protocols involving human subjects. The clinical classification of the participants was based on the Clinical Guidance for Management of Adult COVID-19 Patients advocated by the MoHFW, Government of India (January 2023).

### Next Generation Sequencing (NGS)

COVID–19 positive samples with cycle threshold (Ct) value of <30, were processed, and complementary DNA (cDNA) preparation was done using the SuperScript VILO cDNA Synthesis kit (Invitrogen, Cat # A45003 Thermo Fisher Scientific, USA). SARS-CoV-2 library was prepared using 10 μl of cDNA using a commercial Ion AmpliSeq kit (Ref. A35907) for Chef DL8 (ThermoFisher Scientific, USA). The final library was adjusted to 75 pM before loading onto an Ion Chef instrument for emulsion PCR, enrichment, and subsequently onto an Ion 540 chip. NGS was performed using the Ion Torrent NGS System (Ref. A45003) using an Ion GenStudio S5 Plus System (ThermoFisher Scientific, USA). Raw data were analysed using the Torrent Suite™ software ver.5.12.0, and the NGS QC Toolkit ver.2.3.3 was employed to ward off low-quality and short reads. Variant Caller ver.5.10.1.19 was used to detect variants, compared to the Wuhan-Hu-1 genome (GenBank accession number MN908947.3), and the consensus sequence was developed using IRMAreport ver.1.3.0.2. The annotation was performed using COVID-19AnnotateSnpEff ver.1.3.0.2, a plugin developed explicitly for SARS-CoV-2 to predict the effect of base substitution. Further, the FASTA files were downloaded from Torrent Suite™ software ver.5.12 (ThermoFisher Scientific, USA), and analysed using Pangolin (https://cov-lineages.org/resources/pangolin.html) and NextClade (https://clades.nextstrain.org) software. Sequences were selected with N% <3 and 98.8% mean raw accuracy with the reference genome (Wuhan-Hu-1 strain). The threshold for consensus generation was based on colour coding (as provided in Supplemental Figure S2).

### Phylogenetic analysis

Sequence analysis was performed using Genome Annotation Transfer Utility (GATU) and an ‘in house’ bioinformatics pipeline in Python (available upon request). While there are some mutations (e.g., *A67V*, *Q493R*, *N679K*, *D796Y*) documented in several genes of the virus (e.g., receptor-binding domain (RBD), N-terminal domain (NTD), sub-domain (SD1/2) furin cleavage site, fusion peptide (FP), and heptapeptide repeat (HR1) region), several studies have focused on mutations in the S gene due to comparatively large mutations in the same. Therefore, we chose to conduct phylogenetic analyses towards S gene sequences in the current study. This pipeline was developed to process and translate the sequences rapidly. The sequences were cropped to the S gene of our interest using coordinates provided by National Center for Biotechnology Information (NCBI) and translated to protein sequences automatically through the ExPasy server. After translation, all sequences were mapped with the reference protein sequence using (Multiple Alignment using Fast Fourier Transform) MAFFT ver.7.0 multiple sequence alignment program. The phylogenetic tree was also constructed using the Neighbour-Joining (NJ) method and the Molecular Evolutionary Genetics Analysis software (MEGA X) program with the Maximum Likelihood approach.

### Statistical analysis

The case-patient demography, vaccination status, underlying conditions, viral variants and their association with disease severity, medical support required, and clinical outcome(s) were evaluated univariately using binary regression analysis (presented as a heat map). The variables significantly associated with the clinical outcome by the univariate analysis were subsequently included in the multivariate binary regression (presented as a forest plot (blobbogram) and Table 3). The odds ratio (OR) and 95% CI were estimated. Regression analysis was performed using SPSS software ver.24.0 (SPSS Inc., Chicago, IL, USA). Two-tailed p <0.05 was considered statistically significant for all tests performed. p values <0.05, <0.01, <0.001, <0.0001 were marked as ∗, ∗∗, ∗∗∗ and ∗∗∗∗, respectively.

**Table 1:** Case-patient clinico-demographic characteristics.

Total case-patients, *n*	244
Age, years; *median (IQR)*	42 (29–64.75)
Gender, male; *n (%)*	128 (52.5)
Vaccination status, yes; *n (%)*	198 (81.1)
Type of vaccine; *n (%)*	
AZD1222	141 (57.8)
BBV152	43 (17.6)
BNT162b2	10 (4.1)
mRNA-1273	4 (1.6)
Number of vaccine doses administered; *n (%)*	
One dose	7 (2.9)
Two doses	134 (54.9)
Three or more doses	57 (23.3)
History of COVID-19, yes; *n (%)*	44 (18)
Vaccination prior to COVID-19 disease, yes; *n (%)*	199 (81.6)
Underlying conditions, yes; *n (%)*	81 (33.2)
Severity, *n (%)*	
Asymptomatic	55 (22.6)
Mild	151 (61.9)
Moderate	24 (9.8)
Severe	14 (5.7)
Medical support, *n (%)*	
Hospitalisation	69 (28.3)
Oxygen support	4 (1.6)
HDU/ICU admission	6 (2.5)
Ventilator	0 (0)
Clinical outcomes, *n (%)*	
Terminally ill	6 (2.5)
Recovered	238 (97.5)
SARS-CoV-2 variants, *n (%)*	
XBB	24 (9.8)
XBB.1	56 (23)
XBB.2	20 (8.2)
XBB.3	139 (57)
XBB.5	5 (2)

All data reported as numbers (n) and percentages (%) except for age which is reported as median (IQR).

HDU, high dependency unit; ICU, intensive care unit.

BNT162b2 (Pfizer-BioNTech) is a lipid nanoparticle-formulated, nucleoside-modified mRNA vaccine; mRNA-1273 is an mRNA-based vaccine that contains elasomeran, which instructs the host cell to produce a protein from the original strain of SARS-CoV-2; AZD1222 is an adenoviral vector-based vaccine; BBV152 is a whole-virion-based inactivated vaccine.

**Table 2:** Common spike mutations in different variants and Indian sequences.

A matrix is generated with this identified mutation data in our cohort together with XBB sub-variants, omicron, alpha, beta, gamma, and delta variants of concern to understand the distribution of most prevalent mutations in the S gene. The prevalence of common mutations was plotted with an ‘in house’ R script (the NCBI Reference Sequence: NC_045512.2).

**Table 3:** Factors that predict severity of illness due to infection by an XBB variant of SARS-CoV-2.

Variable	Odds ratio (95% CI)	p value
Age (5)	0.47 (0.26–0.86)	0.015
Vaccination	64.36 (0–140.17)	0.817
AZD1222	3.04 (0.2–45.12)	0.420
No. of vaccine doses	2.79 (0.26–29.94)	0.396
Vaccination prior to COVID-19	0.006 (0–1.35)	0.774

By using binary regression model (please refer to Fig. 2B), variables that showed a significant relationship with the terminal illness in age adjusted model were subsequently included in the multivariate model.

Factors with p <0.05 multivariate model were considered as independent predictors of terminal illness. Age (5)—represent with increase of every 5 years.

### Role of the funding source

The funders of the study had no role in the study design, data collection, data analysis, data interpretation, or writing of the report.

## Results

### Clinico-demographic data and cohort characteristics

The State Public Health Laboratory is equipped with a WGS facility, and is the nodal laboratory for the continuous surveillance and monitoring of newer variants of SARS-CoV-2 in Tamil Nadu. Following a surge in COVID-19 cases during September 2022 with reports of several breakthrough infections and re-infections in the community, we closely monitored the emergence of newer variants of SARS CoV-2. A total of 21,979 COVID-19 cases were reported in Tamil Nadu during September 2022 to January 2023. The peak case load was observed in September 2022 with a test positivity rate of 3.1% followed by a steady decline of COVID-19 cases thereafter (Supplemental Table S8).

**Fig. 1:**
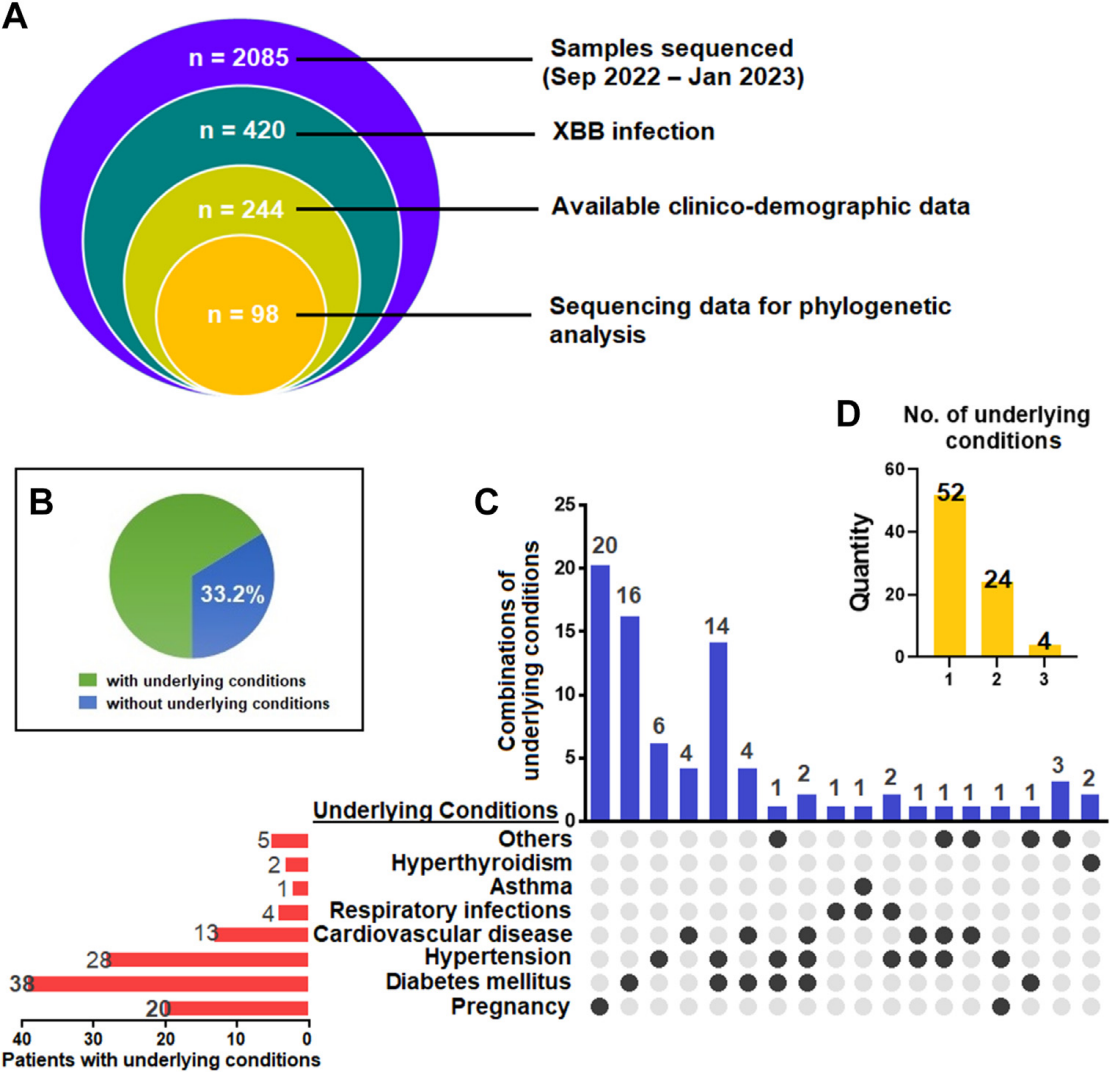
Underlying conditions observed in the patient cohort. **(A)** Venn diagram describing the study cohort. **(B)** Percentage of patients with underlying conditions reported in the cohort. **(C)** Percentage of underlying conditions and in combinations. Others include Parkinson’s disease (n = 2), high haemoglobin levels (polycythaemia) (n = 1), seizure disorders (n = 1), hysterectomy (n = 1) and open-heart surgery (n = 1). **(D)** Number of patients who had reported having one, two and three or more underlying conditions.

Of the 2085 COVID-19 samples sequenced between September 2022 and January 2023, 420 were reported as XBB (20.14%) variants in Tamil Nadu. Of the 420 XBB variants, 244 were selected based on collective information to study the clinico-demography of the cohort (Table 1) (Supplemental Table S1), and 98 were chosen for sequence studies (Supplemental Table S6). A Venn diagram describing the cohort is presented in Fig. 1A. The infected individuals encompassed the following: international travellers, hospitalised individuals, patients admitted in the intensive care unit (ICU)/high dependency unit (HDU), and terminally-ill patients. The sequenced XBB cases (n = 98) encompassed the following sub-lineages: XBB, XBB.1, XBB.2, XBB.3, and XBB.5.

The cohort's median age in the present study was 42 years (Supplemental Table S2A) (IQR = 29–64.75). We also noticed that the XBB variant infected both male and female populations almost equally (% of males = 52.5%), implying no gender-based predilection of the virus (Supplemental Table S2B). Numerically, the cases were high among the 31–50 age group. However, it was observed that the cohort displayed an equal distribution of infection across the age groups. Most cases reported mild (61.9%) clinical outcomes, whereas 22.6% were asymptomatic, and 9.8% had moderate clinical outcomes. The virus also attributed to 28.3% of hospital admissions (n = 69) during the study period. Only 5.7% of the infected had severe disease manifestations requiring ICU/HDU admissions or oxygen support. Six of the severely affected were admitted to the ICU/HDU (6.9%), while four were on oxygen support (5.7%). Among those, XBB.5 was the least prevalent, infecting only five individuals (2.0%); XBB.2 was identified in 20 case-patients (8.2%), XBB was found in 24 individuals (9.8%), XBB.1 was seen in 56 individuals (23%), and XBB.3 seems to be the predominant sub-lineage of XBB, infecting 139 individuals, an alarming 57% of the total cases reported in this cohort. For most people, i.e., 200 of 244, this was their first exposure, while for 44 others this was their second COVID-19 encounter, among which 25 were infected with the XBB.3 variant. Based on the data, XBB.3 appears to be more virulent and prevalent within the study cohort.

Vaccination does not seem to confer protection as 81.6% of the infected people were vaccinated, of which 78.2% were fully vaccinated with two or more doses, and 2.9% were partially vaccinated with a single dose. AZD1222 (57.8%), BBV152 (17.6%), BNT162b2 (4.1%), and mRNA-1273 (1.6%) were preferred by the vaccinated individuals (Supplemental Table S3). It is also interesting to note that while healthcare professionals make up 3% of the infected population, pregnancy constitutes 11% among the infected females. XBB variants have also infected six children <15 years of age, with two hospitalisations. Underlying conditions could increase the risk of infection, as 81 (33.2%) of the infected individuals reported other underlying conditions (Supplemental Table S4A). Most of the participants reported a single underlying condition (n = 52), while a significant number of others reported two (n = 24) or more underlying conditions (Supplemental Table S4B). Diabetes (38%), hypertension (28%), and cardiovascular disease (13%) were the substantial underlying conditions (Fig. 1). The data indicate that omicron sub-variants have substantially evaded the neutralising antibodies generated by COVID-19 vaccination, highlighting the potential need for a bivalent vaccination with a VOC to prevent the surging number of SARS-CoV-2 infections with newer variants.

### XBB disease severity is significantly associated with age

**Fig. 2:**
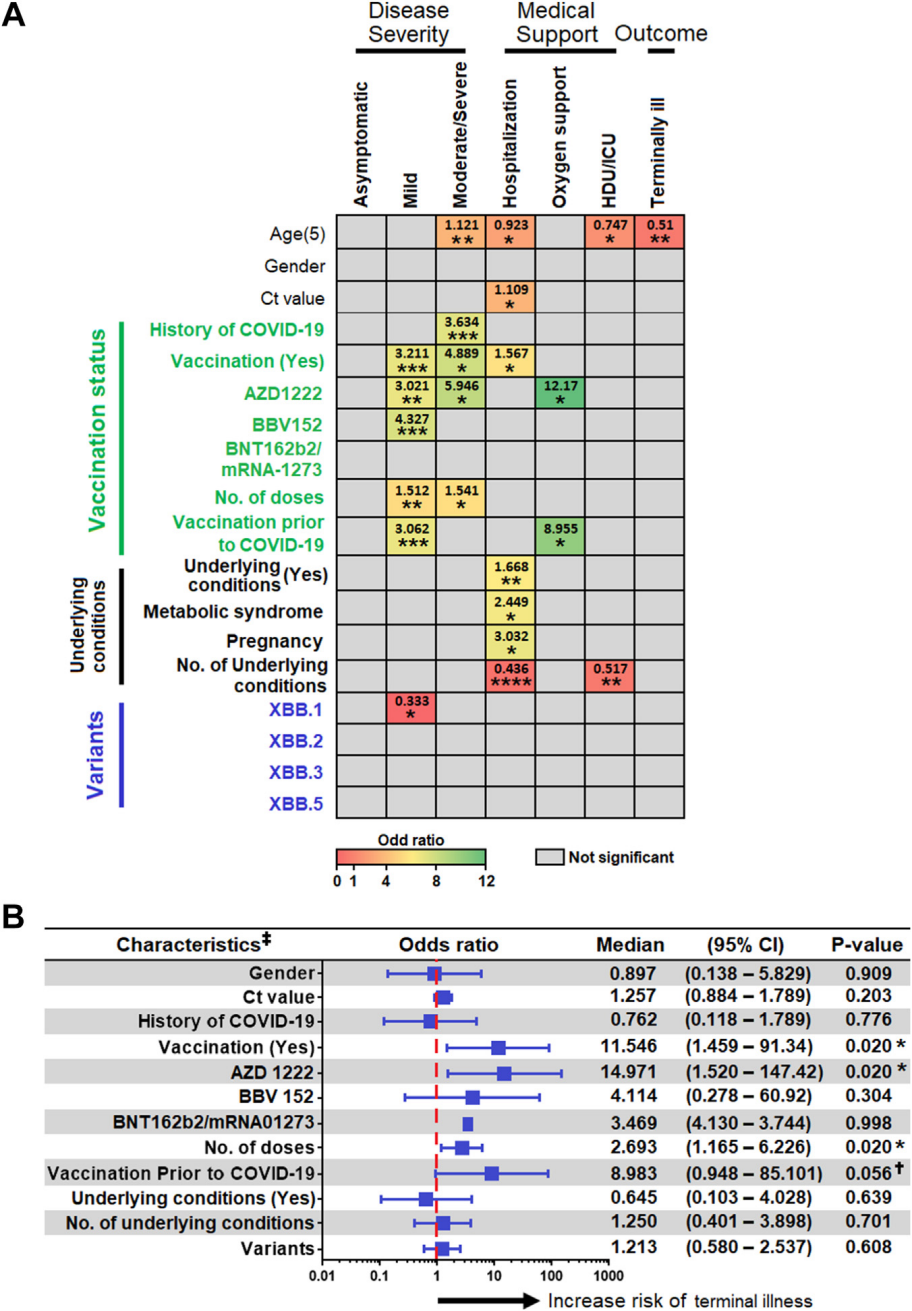
**(A)** Univariate binary regression analysis of factors associated with disease severity, use of medical supports and disease outcome. The odds ratio of a significant association is depicted according to the colour scale. Odds ratio of an insignificant association is depicted in grey. The complete regression analysis results were provided in Supplemental Table S7A–S7G. ∗, ∗∗, ∗∗∗, ∗∗∗∗ represent p < 0.05, <0.01, <0.001 and <0.0001, respectively. **(B)** Association of patients’ characteristics with risk of development of terminal illness. Given that the age was the only variable that associated with terminal illness in univariate analysis, here the regression analysis was performed controlling for age. Footnotes: ∗p < 0.05; ^†^Having a trend of significant associated; ^‡^All variable were adjusted for age. Age (5) represents increase of every 5 years. Ct, cycle threshold; HDU, high dependency unit; ICU, intensive care unit. BNT162b2 (Pfizer-BioNTech) is a lipid nanoparticle-formulated, nucleoside-modified mRNA vaccine; mRNA-1273 is an mRNA-based vaccine that contains elasomeran, which instructs the host cell to produce a protein from the original strain of SARS-CoV-2; AZD1222 is an adenoviral vector-based vaccine; BBV152 is a whole-virion-based inactivated vaccine.

Our analysis showed that the age of acquisition of infection plays a significant role in determining the progression towards development of moderate/severe disease (Supplemental Table S5A), hospitalization, ICU/HDU admission (Supplemental Table S5B), and mortality (OR 1.121, 0.923, 0.747, and 0.51, respectively). While the history of COVID-19 does not have much effect on disease severity, vaccination had a significant association with moderate/severe disease outcomes and hospitalization. Of note, the administration of AZD1222 is strongly related to severe disease manifestations and hospitalisations by 5.946 and 12.17 folds, respectively. Metabolic syndromes and pregnancy had a significant association with hospitalisation. The more the underlying conditions, the greater were the chances of getting hospitalised or admitted to the ICU/HDU (Supplemental Tables S5C and S5D). Of the different XBB sub-lineages identified, XBB.1 was significantly associated with development of mild disease (Fig. 2A).

Given that age was the only variable associated with a terminal illness in the univariate analysis, we performed a regression analysis controlling for age. Vaccination status (whether vaccinated or not), vaccination with the AZD1222 vaccine, number of doses of vaccine administered, and whether the vaccine was taken prior to the exposure of COVID-19 were the four significant variables with p values of 0.02, 0.02, 0.02, and 0.056, respectively, and were associated with an increase in the risk of developing terminal illness (Fig. 2B). In a multivariate analysis, all the five aforesaid variables [viz., age, vaccination status (whether vaccinated or not), administration of AZD1222, number of doses of vaccine administered, and whether the vaccine was taken prior to exposure to COVID-19], only age was independently associated with increased risk of developing terminal illness (Table 3). Overall, these data demonstrate that age plays a critical role in disease severity associated with the incidence of infection with the highly mutated omicron sub-variants.

### Sequence analyses revealed two unique mutations, A27S and T747I, in the XBB variants

**Fig. 3:**
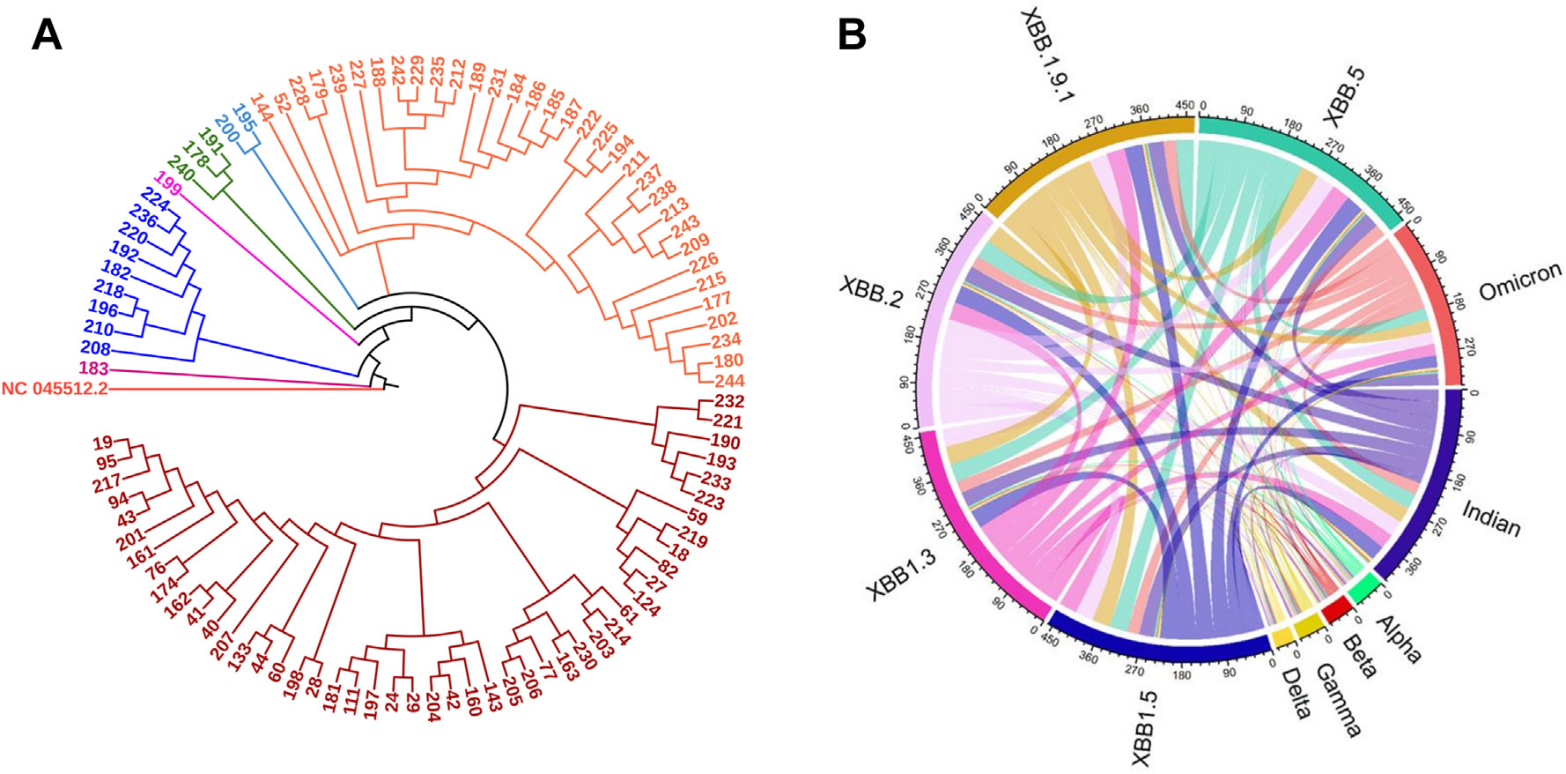
**(A)** The FASTA sequences used in the analysis are acquired from GenBank®. All the sequences are from the XBB variant, and we were interested in finding the variations present in the S gene. All the sequences were mapped to the reference Wuhan-Hu 1 SARS-CoV-2 isolate’s complete genome (NC_045512.2). Sequence analysis was performed using Genome Annotation Transfer Utility (GATU), an ‘in house’ bioinformatics pipeline written in Python (available upon request). This pipeline was developed to rapidly process and translate the sequences. The sequences were cropped to the gene of our interest using coordinates provided by NCBI and translated to protein sequences automatically through the ExPasy server. After translation, all the sequences were mapped with the reference protein sequence using the MAFFT (ver.7.0) multiple sequence alignment program Phylogenetic tree was also constructed using NJ method and MEGA X program with Maximum Likelihood approach. The variation was then called out and deposited as a data file for further interpretation. **(B)** The chord diagram shows the common mutations among different variants of SARS-CoV-2. Majority of mutations are common among XBB sequences (34–40), whereas 2–3 mutations are common between the alpha, beta, gamma and delta variants and the remaining Omicron sub-variants (the details of common mutations are presented in Table 2). Footnotes: FASTA, FastAll; NCBI, GATU, Genome Annotation Transfer Utility; MAFFT, Multiple Alignment using Fast Fourier Transform; MEGA X, Molecular Evolutionary Genetics Analysis software; NCBI, National Center for Biotechnology Information; NJ, Neighbour-Joining.

Sequence analysis showed a distinction among the different sub-lineages of XBB in that XBB.1 had a unique G252V mutation in the S protein. The XBB.3 had a sole mutation I1988V at the ORF 1b, and XBB.5 showed an A653V mutation in the S gene. A total of 43 mutations were identified in the S gene among 98 sequences (Supplemental Table S6). Most mutations were common among XBB sequences (34–40), whereas two to three mutations were shared between the Alpha, Beta, Gamma, and Delta variants and the remaining omicron sub-variants (Fig. 3C, Table 2). Two mutations, A27S and T747I, were unique to the sequences isolated from the patient cohort. These mutations have not been reported before in any of the XBB VOCs reported so far and are believed to be present in regions where vaccine epitopes are present. However, its impact on the clinical outcomes and disease transmissibility warrants further investigation.

Moreover, our phylogenetic analysis showed the presence of six significant branches among the 98 sequences. One patient was infected with a virus (sample ID 28548183) that was the closest relative of the wild-type Wuhan-Hu-1 strain. The rest of the branches originated from the unique (magenta) sequence (Fig. 3B). Additionally, different clusters of patients were remotely related to the wild-type Wuhan-Hu-1 sequence (Fig. 3B) and were branched out, suggesting the evolution of these variants circulating in the population. The distinct phylogenetic branches indicate that the viruses from different patients emerged independently and were unrelated to any particular source or origin. Together, these data revealed the identification of two distinct novel mutations in XBB variants in the southern part of India, which has not been reported elsewhere thus far.

## Discussion

We report identifying these unique mutations in the XBB omicron variant viz., T981S, I1998V, T323I, G252V, A653V, and two novel mutations of A27S and T747I, which have not been reported from any other XBB VOCs till now. The possible difference in morbidity between survivors and the terminally ill cases reported herein could have resulted from a novel mutation identified in the viral genome, likely resulting in a significant degree of immune evasion and infectiousness observed among terminally-ill cases. Earlier studies have already demonstrated the effect of mutations in inducing immune escape of the virus. Antibody binding to the S protein appears significantly affected by mutations in the immune escape hotspots E484, F486, and F490 residues.^17^^,^^18^ The S477N and P681H mutations play an essential role in SARS-CoV-2 infectivity by enhancing its binding affinity for ACE2,^19^ and by augmenting furin cleavage of the S protein,^20^ respectively. H655Y is a mutation in the non-RBD of the S protein that can independently enhance the binding of the S protein with ACE2 or in combination with the N501Y mutation.^21^

Early clinical findings with the omicron variant showed only mild symptoms such as myalgia, fatigue, rhinorrhoea, and others with varying symptomatic and asymptomatic forms.^22^ Nonetheless, given the various mutations, especially in the S protein,^23^ there were mounting concerns about the expanded contagiousness and escape of the variant from vaccine-mediated or infection-induced immune responses.^22^^,^^24^ The notoriety of the omicron variant continued to increase further with the emergence of variants of omicron, especially BA.1.1 and BA.2, which was able to evade immunity generated by vaccination or prior exposure.^25^ The derivative BA.1.1 was distinguished from the original omicron strain with an additional R346K mutation, which showed decreased neutralisation against antibodies generated by two-dose vaccinations. Still a third booster dose regimen was found to stand a chance against the virus. However, the BA.2 and BA.3 strains continued to have evasion abilities against antibodies generated even after a third dose.^26^ Nick-named the ‘stealth omicron’, the BA.2 lineage contrasts the “standard” omicron lineages by not having the SGTF-causing deletion (H69del and V70del).^27^ The BA.4 and BA.5 strains also showed evidence of immunological escape from the vaccine/BA.1 infection response, the vulnerability increases in the months after vaccination or infection owing to the waning of responses over time.^28^^,^^29^ Another study by Kurhade and colleagues has also summarised that the recently discovered omicron sub-lineages are more immune evasive to vaccination and/or infection-induced neutralisation. BA.2.75 is one of the three well-studied omicron sub-lineages with the most significant resistance to vaccine-induced neutralisation. Reporting of BA.2.75.2, BQ.1.1, and XBB.1 suggests that these novel sub-lineages can become the predominant lineage in circulation.^30^ A similar observation on the XBB variant’s high immune escape potential was also reported from the state of Maharashtra (a southwestern state in India) as well.^31^ These reports align with the findings of our study that the XBB variant may have the upper hand in infectivity compared to the other strains mainly due to the accumulation of mutations from the two parental strains BA.2.10.1 and BA.2.75 and due to novel mutations in the strain. Considering the likelihood of increased infectivity and pathogenicity of the XBB variants, current literature suggests that thermodynamic driving forces of viral binding and biosynthesis would have key implications on the aforesaid attributes^32^ because any change in Gibbs energy of antigen-receptor binding would enhance viral infectivity.^33^ Furthermore, Gibbs energy of virus biosynthesis is the major driving force for any change in viral pathogenicity.^34^ Gibbs energy of virus binding has been elucidated for many variants of SARS-CoV-2, and hence, investigating the thermodynamic components is warranted to understand the complexities of host-virus interactions and virus evolution. Moreover, an increased disease burden can be predicted even among the vaccinated and/or prior infected population.

Our cohort study has a unique strength where using a comprehensive regression analysis, we evaluated the omicron variants, underlying conditions, and their association with disease severity, medical support required as well as treatment outcome. This has allowed continuous surveillance of omicron variants and systematic evaluation of their clinical impact on the population. Further, our cross-sectional cohort study also permitted the continuous surveillance of oropharyngeal and nasopharyngeal samples retrieved from across the entire state of Tamil Nadu to the State Public Health Laboratory for comprehensive NGS investigations that has led to the detection of different XBB sublineages. More importantly, our genomic surveillance measures have helped the public health system to develop appropriate policy decisions pertinent to the effective control of the COVID-19 pandemic.

Our study has some limitations. The current investigation was restricted to the regions where the public health laboratory had access to samples and did not sample large number of infections in the given areas. Furthermore, the distribution of study samples may have had a bias largely owing to a restricted geographic locality rather than widespread areas across the entire southern India. Therefore, future studies are warranted to precisely delineate the origin and transmission of these variants in the community. Moreover, we did not perform neutralisation studies using the actively circulating variants of SARS-CoV-2 and sera from COVID-19 positive individuals to further comprehend our findings of immune evasion by the identified XBB sub-lineages of the virus. The high rate of breakthrough infections (82%) observed herein attributed to the XBB variants should be regarded as a likely component of viral virulence irrespective of the clinical outcome. Moreover, our limited data cannot effectively deduce whether the newly evolved XBB variant can cause significant mortality. Furthermore, the impact of the unique mutations on clinical course of illness and disease transmissibility warrants further investigation as it may be premature to draw conclusions based on the sequence data generated herein.

In conclusion, our observations indicate the identification of highly immune evasive virulent sub-lineages of the XBB variant. Previous infection or vaccination does not guarantee protection owing to the high frequency of mutations in its genome. Nonetheless, precedence has taught us that vaccination is the practical option for protecting the population at large by conferring herd immunity, which also appears to be the best prophylactic choice available for the vulnerable population.^12^^,^^33^ Our findings advocate the global need for the continuous genomic surveillance of SARS CoV-2 to closely monitor the emergence of new variants in the community. Given the evolution of new sublineages (like the XBB variants), we also concur with global unanimity that the COVID-19 threat is far from over. For instance, due to the recent emergence of omicron subvariant EG.5 (a descendent lineage of XBB.1.9.2), countries in the West Pacific region, USA, UK, France, and Japan are experiencing a surge in COVID-19 cases.^35^ Our report of >80% of breakthrough infections highlight the urgent need for polyvalent vaccines. Our findings reveal the importance of understanding viral evolution pertinent to disease transmission and revamping the current treatment options following the continuous evolution of viral strains. Our findings also advocate the need for bivalent or bivalent booster vaccination for the entire population to protect them from emerging VOCs.

## Contributors

STS, YKY, NJ, AM, ML, SNB, VV, EMS, and SR were responsible for conceptualization and data curation. STS, SJK, NJ, KH, YKY, SS, VK, RV, SG, MR, AK, SC, BA, NG, ARA, BG, HB, PJ, MK, AM, SLK, KS, SP, ML, SLK, KS, SNB, VV, EMS, and SR were responsible for methodology, formal analysis, validation, and visualization. STS, YKY, NJ, ML, SG, KS, SNB, VV, EMS, and SR were responsible for the original draft, while all authors reviewed, edited, and approved the final manuscript. All authors had full access to all data in the study and take responsibility for the decision to submit for publication.

## Data sharing statement

The genome sequences in the study are openly available in GenBank with reference numbers OQ569699–OQ569715, OQ587517–OQ587574, OQ652056, OQ652057, OQ652066, OQ652067, OQ652069, OQ652070, OQ652149, OQ652150, OQ652151, OQ652152, OQ652153. The de-identified clinical data of patients in the study and ‘in house’ bioinformatics pipeline in Python are available on request to the corresponding author.

## Declaration of interests

STS and SR are funded by the National Health Mission, Tamil Nadu (680/NGS/NHMTNMSC/ENGG/2021) for the Directorate of Public Health and Preventive Medicine, WGS facility. EMS is supported by the Indian Council of Medical Research (ICMR) Grant No. 45/2/2020-DDI/BMS and a Core Research Grant (CRG) of the Department of Science and Technology-Science and Engineering Research Board (DST-SERB), Government of India (File No. CRG/2019/006096). ML is supported by grants through AI52731, the Swedish Research Council, the Swedish, Physicians against AIDS Research Foundation, the Swedish International Development Cooperation Agency, SIDA SARC, VINNMER for Vinnova, Linköping University Hospital Research Fund, CALF, and the Swedish Society of Medicine. VV is supported by the Office of Research Infrastructure Programs (ORIP/NIH) base grant P51 OD011132 to ENPRC. AM is supported by a Start-up Grant No. 12020/04/2018-HR, Department of Health Research, Government of India. The authors declare that there are no other relationships or activities that might bias, or be perceived to bias, their work.
