# Supplementary material for: Clinical characteristics and novel mutations of omicron subvariant XBB in Tamil Nadu, India – a cohort study
Source: Lancet Reg Health Southeast Asia. 2023 Sep 4;19:100272. doi: 10.1016/j.lansea.2023.100272 (PMC10709680; doi:10.1016/j.lansea.2023.100272)
Supplement: Supplemental Tables S1–S8 and Figures S1–S2 [file mmc1.pdf]

**Novel mutations in XBB variants of SARS-CoV-2 decipher high rates of morbidity among COVID-19 patients in South India**

**Supplemental Table 1:** Regional distribution of SARS-CoV-2 Omicron XBB sub-lineages (n=244).

| S. No | District | Omicron XBB sub-variants | | | | | Total |
|---|---|---|---|---|---|---|---|
| | | XBB | XBB.1 | XBB.2 | XBB.3 | XBB.5 | |
| 1 | Chennai | 10 | 29 | 7 | 77 | 0 | 123 |
| 2 | Coimbatore | 0 | 6 | 3 | 9 | 0 | 18 |
| 3 | Kanchipuram | 0 | 3 | 1 | 8 | 1 | 13 |
| 4 | Tiruvannamalai | 0 | 2 | 0 | 10 | 0 | 12 |
| 5 | Erode | 0 | 1 | 1 | 10 | 0 | 12 |
| 6 | Thiruvallur | 1 | 1 | 0 | 6 | 0 | 8 |
| 7 | Chengalpattu | 0 | 2 | 3 | 2 | 0 | 7 |
| 8 | Tiruchirappalli | 1 | 2 | 0 | 2 | 1 | 6 |
| 9 | Madurai | 3 | 1 | 0 | 1 | 0 | 5 |
| 10 | Salem | 0 | 3 | 1 | 0 | 1 | 5 |
| 11 | Villupuram | 1 | 1 | 0 | 2 | 0 | 4 |
| 12 | Perambalur | 1 | 0 | 0 | 1 | 2 | 4 |
| 13 | Ranipet | 1 | 0 | 0 | 3 | 0 | 4 |
| 14 | Ramanathapuram | 2 | 0 | 0 | 1 | 0 | 3 |
| 15 | Tirunelveli | 1 | 0 | 0 | 2 | 0 | 3 |
| 16 | Kanyakumari | 1 | 1 | 1 | 0 | 0 | 3 |
| 17 | Tiruppur | 0 | 0 | 1 | 1 | 0 | 2 |
| 18 | Dindigul | 1 | 0 | 0 | 1 | 0 | 2 |
| 19 | Vellore | 1 | 1 | 0 | 0 | 0 | 2 |
| 20 | Pudukottai | 0 | 0 | 1 | 0 | 0 | 1 |
| 21 | Virudhunagar | 0 | 0 | 0 | 1 | 0 | 1 |
| 22 | Thanjavur | 0 | 0 | 0 | 1 | 0 | 1 |
| 23 | The Nilgiris | 0 | 0 | 1 | 0 | 0 | 1 |
| 24 | Thoothukudi | 0 | 1 | 0 | 0 | 0 | 1 |
| 25 | Theni | 0 | 1 | 0 | 0 | 0 | 1 |
| 26 | Ariyalur | 0 | 0 | 0 | 1 | 0 | 1 |
| 27 | Thiruvarur | 0 | 1 | 0 | 0 | 0 | 1 |
| | **Grand total** | **24** | **56** | **20** | **139** | **5** | **244** |

**Supplemental Table 2A:** Age-wise distribution of SARS-CoV-2 Omicron XBB sub-lineages (n=244)

| **Age group (in years)** | **Omicron XBB sub-variants** | | | | | | **Percentage** |
|---|---|---|---|---|---|---|---|
| | **XBB** | **XBB.1** | **XBB.2** | **XBB.3** | **XBB.5** | **Total** | |
| 0-12 | 0 | 2 | 0 | 3 | 0 | 5 | **2.04** |
| 13-18 | 0 | 2 | 0 | 4 | 0 | 6 | **2.45** |
| 19-30 | 06 | 11 | 4 | 35 | 2 | 58 | **23.77** |
| 31-50 | 7 | 20 | 5 | 44 | 2 | 77 | **31.55** |
| 51-65 | 5 | 10 | 4 | 22 | 0 | 41 | **16.8** |
| >65 | 6 | 12 | 7 | 31 | 1 | 57 | **23.36** |
| **Total** | **24** | **56** | **20** | **139** | **5** | **244** | **100** |
| **Percentage** | **9.8** | **23** | **8.2** | **57** | **2** | **100** | |

**Supplemental Table 2B:** Gender-wise distribution of SARS-CoV-2 Omicron XBB sub-lineages (n=244)

| **Age group** | **Omicron XBB sub-variants** | | | | | | **Percentage** |
|---|---|---|---|---|---|---|---|
| | **XBB** | **XBB.1** | **XBB.2** | **XBB.3** | **XBB.5** | **Total** | |
| Male | 13 | 31 | 12 | 69 | 3 | 128 | 52 |
| Female | 11 | 25 | 8 | 70 | 2 | 116 | 48 |
| Total | 24 | 56 | 20 | 139 | 5 | 244 | 100 |
| **Percentage** | **9.8** | **23** | **8.2** | **57** | **2** | **100** | |

**Supplemental Table 3:** Type of vaccines administered in the study group (n=244)

| Vaccine ID | Proprietary name | Manufacturer | Type of vaccine | Mode of Administration | Characteristics | No. of cases | Percentage |
|---|---|---|---|---|---|---|---|
| AZD1222 | Covishield™ | Serum Institute of India Pvt Ltd and COVID-19 vaccine AstraZeneca (AstraZeneca) | Recombinant | Intramuscular injection | Recombinant, replication-deficient chimpanzee adenovirus vector encoding the SARS-CoV-2 spike (S) glycoprotein | 141 | 58 |
| BBV152 | Covaxin® | Bharath Biotech | Inactivated viron | Intramuscular injection | β-propiolactone inactivated vaccine | 43 | 18 |
| BNT162B2 | Pfizer-BioNTech | Pfizer and BioNTech | Messenger RNA (mRNA) vaccine | Intramuscular injection | Messenger RNA (mRNA) vaccine uses a genetic code called RNA to make your body's cells produce the coronavirus' specific spike protein | 10 | 4 |
| mRNA1273 | Spikevax | American company Moderna, the United States National Institute of Allergy and Infectious Diseases (NIAID), and the Biomedical Advanced Research and Development Authority (BARDA) | Nucleoside-modified mRNA (modRNA) | Intramuscular injection | Lipid nanoparticle–encapsulated mRNA-based vaccine | 4 | 2 |
| Not vaccinated | | | | | | 44 | 18 |
| Unknown | | | | | | 2 | 1 |
| **Total** | | | | | | **244** | |

**Supplemental Table 4A:** Underlying conditions among individuals infected with SARS-CoV-2 Omicron XBB sub-lineages (n=244)

| S. No | Underlying condition | Omicron XBB sub-variants | | | | | Total | Percentage |
|---|---|---|---|---|---|---|---|---|
| | | XBB | XBB.1 | XBB.2 | XBB.3 | XBB.5 | | |
| 1 | Diabetes mellitus | 5 | 8 | 4 | 21 | 0 | 38 | 15.6 |
| 2 | Hypertension | 4 | 5 | 3 | 17 | 1 | 30 | 12.3 |
| 3 | Cardiovascular disease | 1 | 1 | 0 | 8 | 2 | 12 | 4.9 |
| 4 | Thyroid dysfunction | 0 | 1 | 0 | 2 | 0 | 3 | 1.2 |
| 5 | Hyponatraemia | 0 | 0 | 0 | 2 | 0 | 2 | 0.8 |
| 6 | Parkinson's disease | 0 | 0 | 0 | 2 | 0 | 2 | 0.8 |
| 7 | Asthma | 0 | 0 | 0 | 2 | 0 | 2 | 0.8 |
| 8 | Arthritis | 0 | 1 | 0 | 0 | 0 | 1 | 0.4 |
| 9 | Sinusitis | 0 | 0 | 0 | 1 | 0 | 1 | 0.4 |
| 10 | Seizures | 0 | 0 | 0 | 0 | 1 | 1 | 0.4 |
| 11 | Polycythemia | 1 | 0 | 0 | 0 | 0 | 1 | 0.4 |
| 12 | Chronic liver disease | 0 | 0 | 0 | 0 | 1 | 1 | 0.4 |
| 13 | Lower respiratory infection | 0 | 0 | 0 | 1 | 0 | 1 | 0.4 |
| 14 | Pneumonia | 0 | 0 | 1 | 0 | 0 | 1 | 0.4 |
| 15 | Upper respiratory infection | 0 | 0 | 0 | 1 | 0 | 1 | 0.4 |
| 16 | No underlying condition | 15 | 43 | 15 | 101 | 3 | 177 | 73 |
| 17 | Unknown | 0 | 2 | 0 | 0 | 0 | 2 | 0.8 |

**Supplemental Table 4B:** Underlying conditions among individuals infected with SARS-CoV-2 Omicron XBB sub-lineages (n=244)

| S. No | Underlying condition | Omicron XXB sub-variants | | | | | Total | Percentage |
|---|---|---|---|---|---|---|---|---|
| | | XBB | XBB.1 | XBB.2 | XBB.3 | XBB.5 | | |
| 1 | Diabetes mellitus (DM) | 3 | 3 | 1 | 10 | 0 | 17 | 7 |
| 2 | DM+hypertension (HTN) | 1 | 4 | 3 | 7 | 0 | 15 | 6.1 |
| 3 | HTN | 3 | 1 | 0 | 5 | 0 | 9 | 3.7 |
| 4 | Cardiovascular disease (CVD) | 0 | 0 | 0 | 4 | 0 | 4 | 1.6 |
| 5 | DM+CVD | 1 | 1 | 0 | 0 | 0 | 2 | 0.8 |
| 6 | DM+HTN+CVD | 0 | 0 | 0 | 2 | 0 | 2 | 0.8 |
| 7 | HTN+CVD | 0 | 0 | 0 | 2 | 0 | 2 | 0.8 |
| 8 | Thyroid dysfunction (TD) | 0 | 1 | 0 | 1 | 0 | 2 | 0.8 |
| 9 | Arthritis | 0 | 1 | 0 | 0 | 0 | 1 | 0.4 |
| 10 | Asthma+sinusitis | 0 | 0 | 0 | 1 | 0 | 1 | 0.4 |
| 11 | CVD+seizures | 0 | 0 | 0 | 0 | 1 | 1 | 0.4 |
| 12 | DM+hyponatremia | 0 | 0 | 0 | 1 | 0 | 1 | 0.4 |
| 13 | DM+TD+asthma | 0 | 0 | 0 | 1 | 0 | 1 | 0.4 |
| 14 | Polycythemia | 1 | 0 | 0 | 0 | 0 | 1 | 0.4 |
| 15 | HTN+CVD+ chronic liver disease (CLD) | 0 | 0 | 0 | 0 | 1 | 1 | 0.4 |
| 16 | HTN+Lower respiratory infection (LRI) | 0 | 0 | 0 | 1 | 0 | 1 | 0.4 |
| 17 | Parkinsons disease (PD) | 0 | 0 | 0 | 1 | 0 | 1 | 0.4 |
| 18 | PD+hyponatraemia | 0 | 0 | 0 | 1 | 0 | 1 | 0.4 |
| 19 | Pneumonia | 0 | 0 | 1 | 0 | 0 | 1 | 0.4 |
| 20 | Upper respiratory infection (URI) | 0 | 0 | 0 | 1 | 0 | 1 | 0.4 |
| 21 | No underlying condition | 15 | 43 | 15 | 101 | 3 | 177 | 72.5 |
| 22 | Unknown | 0 | 2 | 0 | 0 | 0 | 2 | 0.8 |
| | **Grand total** | **24** | **56** | **20** | **139** | **5** | **244** | **100** |

**Supplemental Table 5A:** Severity of illness in SARS-CoV-2 Omicron sub-variant infected individuals (n=244)

| Clinical illness | Omicron XBB sub-variants reported | | | | | | Percentage |
|---|---|---|---|---|---|---|---|
| | XBB | XBB.1 | XBB.2 | XBB.3 | XBB.5 | Total | |
| Asymptomatic | 5 | 14 | 4 | 31 | 1 | 55 | 22.6 |
| Mild | 15 | 31 | 12 | 91 | 2 | 151 | 61.9 |
| Moderate | 1 | 7 | 3 | 13 | 0 | 24 | 9.8 |
| Severe | 3 | 4 | 1 | 4 | 2 | 14 | 5.7 |
| Total | 24 | 56 | 20 | 139 | 5 | 244 | 100 |

**Supplemental Table 5B:** Clinical course of illness in SARS-CoV-2 Omicron sub-variant infected individuals (n=244)

| Clinical illness | Omicron XBB sub-variants reported | | | | | | Percentage |
|---|---|---|---|---|---|---|---|
| | XBB | XBB.1 | XBB.2 | XBB.3 | XBB.5 | Total | |
| **Hospitalization** | **7** | **12** | **6** | **40** | **4** | **69** | **28%** |
| Without $O_2$ support | 6 | 11 | 5 | 39 | 4 | 65 | 94% |
| With $O_2$ support | 1 | 1 | 1 | 1 | 0 | 4 | 6% |

**Supplemental Table 5C:** Clinical course of illness in SARS-CoV-2 Omicron sub-variant infected individuals (n=244)

| Clinical illness | Omicron XBB sub-variants reported | | | | | | Percentage |
|---|---|---|---|---|---|---|---|
| | XBB | XBB.1 | XBB.2 | XBB.3 | XBB.5 | Total | |
| **Hospitalization** | **7** | **12** | **6** | **40** | **4** | **69** | 28% |
| Without ICU/HDU support | 6 | 11 | 6 | 38 | 2 | 63 | 91% |
| With ICU/HDU support | 1 | 1 | 0 | 2 | 2 | 6 | 9% |

**Supplemental Table 5D:** Clinical course of illness in SARS-CoV-2 Omicron sub-variants infected individuals (n=244)

| Clinical illness | Omicron XBB sub-variants reported | | | | | |
|---|---|---|---|---|---|---|
| | **XBB** | **XBB.1** | **XBB.2** | **XBB.3** | **XBB.5** | **Total** |
| **Hospitalization** | 0 | 0 | 0 | 1 (Mild) | 0 | 1 |
| With $O_2$ support | 1 (severe) | 0 | 0 | 0 | 0 | 1 |
| Under ICU/HDU care | 0 | 1 (asymptomatic) | 0 | 0 | 0 | 1 |
| No hospitalization | 0 | 0 | 1 (asymptomatic) | 2 (1 asymptomatic & 1 moderate) | 0 | 3 |
| **Total** | 1 | 1 | 1 | 3 | 2 | 6 |

**Supplemental Table 6:** Characteristics of mutation detected from SARS-CoV-2 sub-lineages of XBB (including two unique mutations (n=98) highlighted in yellow and green).

| Sample ID | GeneBank Accession ID | Sub-lineages reported | Mutations detected |
|---|---|---|---|
| 18 | OQ587517 | **XBB.3** | M:Q19E, M:A63T, N:P13L, N:R203K, N:G204R, N:S413R, ORF1a:K47R, ORF1a:G82D, ORF1a:S135R, ORF1a:T842I, ORF1a:G1307S, ORF1a:L3027F, ORF1a:T3090I, ORF1a:L3201F, ORF1a:T3255I, ORF1a:P3395H, ORF1b:P314L, ORF1b:G662S, ORF1b:S959P, ORF1b:R1315C, ORF1b:I1566V, ORF1b:I1998V, ORF1b:T2163I, ORF3a:T223I, ORF6:D61L, ORF9b:P10S, S:T19I |
| 19 | OQ569687 | **XBB.3** | E:T11A, M:Q19E, M:A63T, N:P13L, N:R203K, N:G204R, N:S413R, ORF1a:K47R, ORF1a:G82D, ORF1a:S135R, ORF1a:T842I, ORF1a:G1307S, ORF1a:L3027F, ORF1a:T3090I, ORF1a:L3201F, ORF1a:T3255I, ORF1a:P3395H, ORF1b:P314L, ORF1b:G662S, ORF1b:S959P, ORF1b:R1315C, ORF1b:I1566V, ORF1b:I1998V, ORF1b:T2163I, ORF3a:T223I, ORF6:D61L, ORF9b:P10S, S:T19I, S:**A27S**, S:F86I, S:G142D, S:H146Q, S:Q183E, S:V213E, S:G339H, S:R346T, S:L368I, S:S371F, S:S373P, S:S375F, S:T376A, S:D405N, S:R408S, S:K417N, S:N440K, S:V445, S:G446S, S:N460K, S:S477N, S:T478K, S:E484A, S:F486S, S:F490S, S:Q498R, S:D614G, S:H655Y, S:N679KF, S:P681H, S:N764K, S:D796Y, S:Q954H |
| 24 | OQ569688 | **XBB.3** | E:T11A, M:Q19E, M:A63T, N:P13L, N:R203K, N:G204R, N:S413R, ORF1a:K47R, ORF1a:G82D, ORF1a:S135R, ORF1a:T842I, ORF1a:G1307S, ORF1a:L3027F, ORF1a:T3090I, ORF1a:L3201F, ORF1a:T3255I, ORF1a:P3395H, ORF1b:P314L, ORF1b:G662S, ORF1b:S959P, ORF1b:R1315C, ORF1b:I1566V, ORF1b:I1998V, ORF1b:T2163I, ORF3a:T223I, ORF6:D61L, ORF9b:P10S, S:T19I, S:**A27S**, S:G142D, S:H146Q, S:Q183E, S:V213E, S:D215Y, S:G339H, S:R346T, S:L368I, S:S371F, S:S373P, S:S375F, S:T376A, S:D405N, S:R408S, S:K417N, S:N440K, S:V445P, S:G446S, S:N460K, S:S477N, S:T478K, S:E484A, S:F486S, S:F490S, S:Q498R, S:N501Y, S:Y505H, S:D614G, S:H655Y, S:N679K, S:P681H, S:N764K, S:D796Y, S:Q954H |
| 27 | OQ587518 | **XBB.5** | E:T11A, M:Q19E, M:A63T, N:P13L, N:R203K, N:G204R, N:T245I, N:S413R, ORF1a:K47R, ORF1a:G82D, ORF1a:S135R, ORF1a:A599V, ORF1a:T708I, ORF1a:T842I, ORF1a:G1307S, ORF1a:I2010V, ORF1a:L3027F, ORF1a:T3090I, ORF1a:L3201F, ORF1a:T3255I, ORF1a:P3395H, ORF1a:D3668Y, ORF1b:P314L, ORF1b:G662S, ORF1b:S959P, ORF1b:R1315C, ORF1b:I1566V, ORF1b:T2163I, ORF3a:T223I, ORF6:D61L, ORF9b:P10S, S:T19I, S:**A27S**, S:N81R, S:L84F, S:Y91C, S:G142D, S:H146Q, S:Q183E, S:V213E, S:G339H, S:R346T, S:L368I, S:S371F, S:S373P, S:S375F, S:T376A, S:D405N, S:R408S, S:K417N,S:N440K, S:V445P, S:G446S, S:N460K, S:S477N, S:T478K, S:E484A, S:F486S, S:F490S, S:Q498R, S:N501Y, S:Y505H, S:D614G, S:A653V,S:H655Y, S:N679K, S:P681H, S:N764K, S:D796Y, S:Q954H |
| 28 | OQ569689 | **XBB.3** | E:T11A, M:Q19E, M:A63T, N:P13L, N:R203K, N:G204R,N:S413R, ORF1a:K47R, ORF1a:G82D, ORF1a:S135R, ORF1a:T842I, ORF1a:G1307S, ORF1a:A2129S, ORF1a:L3027F, ORF1a:T3090I, ORF1a:L3201F, ORF1a:T3255I, ORF1a:P3395H, ORF1b:P314L, ORF1b:G662S, ORF1b:S959P, ORF1b:R1315C, ORF1b:I1566V, ORF1b:I1998V, ORF1b:T2163I, ORF3a:T223I, ORF6:D61L, ORF7a:A105V, ORF9b:P10S, S:T19I, S:**A27S**, S:G142D, S:H146Q, S:Q183E, S:V213E, S:G339H, S:R346T, S:L368I, S:S371F, S:S373P, S:S375F, S:T376A, S:D405N, S:R408S, S:K417N, S:N440K, S:V445P, S:G446S, S:N460K, S:S477N, S:T478K, S:E484A, S:F486S, S:F490S, S:Q498R, S:N501Y, S:Y505H, S:D614G, S:H655Y, S:N679K, S:P681H, S:N764K, S:D796Y, S:Q954H |
| 29 | OQ587519 | **XBB.3** | E:T11A, M:Q19E, M:A63T, N:P13L, N:R203K, N:G204R, N:S413R, ORF1a:K47R, ORF1a:G82D, ORF1a:S135R, ORF1a:T842I, ORF1a:G1307S, ORF1a:L3027F, ORF1a:T3090I, ORF1a:L3201F, ORF1a:T3255I, ORF1a:P3395H, ORF1b:P314L, ORF1b:G662S, ORF1b:S959P, ORF1b:R1315C, ORF1b:I1566V, ORF1b:I1998V, ORF1b:T2163I, ORF3a:T223I, ORF6:D61L, ORF9b:P10S, S:T19I |
| 40 | OQ569690 | **XBB.3** | E:T11A, M:Q19E, M:A63T, M:H155Y, N:P13L, N:R203K, N:G204R, N:S413R, ORF1a:K47R, ORF1a:G82D, ORF1a:S135R, ORF1a:T842I, ORF1a:G1307S, ORF1a:L3027F, ORF1a:T3090I, ORF1a:L3201F, ORF1a:T3255I, ORF1a:P3395H, ORF1b:P314L, ORF1b:G662S, ORF1b:S959P, ORF1b:R1315C, ORF1b:I1566V, ORF1b:I1998V, ORF1b:T2163I, ORF3a:T223I, ORF6:D61L, ORF9b:P10S, S:T19I, S:**A27S**, S:G142D, S:H146Q, S:Q183E, S:V213E, S:G339H, S:R346T, S:L368I, S:S371F, S:S373P, S:S375F, S:T376A, S:D405N, S:R408S, S:K417N, S:N440K, S:V445P, S:G446S, |

| | | | |
|---|---|---|---|
| | | | S:N460K, S:S477N, S:T478K, S:E484A, S:F486S, S:F490S, S:Q498R, S:N501Y, S:Y505H, S:D614G, S:H655Y, S:N679K, S:P681H, S:N764K, S:D796Y, S:Q954H |
| 41 | OQ587520 | **XBB.3** | E:T11A, M:Q19E, M:A63T, N:P13L, N:R203K, N:G204R, N:S413R,,ORF1a:K47R, ORF1a:G82D, ORF1a:S135R, ORF1a:T842I, ORF1a:G1307S, ORF1a:L3027F, ORF1a:T3090I, ORF1a:L3201F, ORF1a:T3255I, ORF1a:P3395H, ORF1b:P314L, ORF1b:G662S, ORF1b:S959P, ORF1b:R1315C, ORF1b:I1566V, ORF1b:I1998V, ORF1b:T2163I, ORF3a:T223I, ORF6:D61L, ORF9b:P10S, S:T19I |
| 42 | OQ587521 | **XBB.3** | E:T11A, M:Q19E, M:A63T, N:P13L, ORF1a:K47R, ORF1a:S135R, ORF1a:T842I, ORF1a:G1307S, ORF1a:L3027F, ORF1a:T3090I, ORF1a:L3201F, ORF1a:T3255I, ORF1a:P3395H, ORF1b:P314L, ORF1b:G662S, ORF1b:S959P, ORF1b:R1315C, ORF1b:I1566V, ORF1b:I1998V, ORF1b:T2163I, ORF3a:T223I, ORF6:D61L, ORF9b:P10S, S:T19I, S:**A27S**, S:V83A, S:F86L, S:G142D |
| 43 | OQ587522 | **XBB.3** | E:T11A,M:Q19E, M:A63T, N:P13L, N:R203K, N:G204R, N:S413R, ORF1a:K47R, ORF1a:G82D, ORF1a:S135R, ORF1a:T842I, ORF1a:G1307S, ORF1a:H2357Y, ORF1a:L3027F, ORF1a:T3090I, ORF1a:L3201F, ORF1a:T3255I, ORF1a:P3395H, ORF1b:P314L, ORF1b:G662S, ORF1b:S959P, ORF1b:R1315C, ORF1b:I1566V, ORF1b:I1998V, ORF1b:T2163I, ORF3a:T223I, ORF6:D61L, ORF9b:P10S, S:T19I |
| 44 | OQ652066 | **XBB.3** | E:T11A, M:Q19E, N:P13L, N:A152V, N:S187L, N:R203K, N:G204R, N:S413R, ORF1a:K47R, ORF1a:G82D, ORF1a:S135R, ORF1a:T842I, ORF1a:G1307S, ORF1a:L3027F, ORF1a:T3090I, ORF1a:L3201F, ORF1a:T3255I, ORF1a:P3395H, ORF1b:P314L, ORF1b:G662S, ORF1b:S959P, ORF1b:R1315C, ORF1b:I1566V, ORF1b:I1998V, ORF1b:T2163I, ORF3a:T223I, ORF6:D61L, ORF9b:P10S, S:T19I |
| 52 | OQ569691 | **XBB.1** | E:T11A, M:Q19E, M:A63T, N:P13L, N:R36Q, N:R203K, N:G204R, N:S413R, ORF1a:K47R, ORF1a:S135R, ORF1a:T842I, ORF1a:G1307S, ORF1a:L3027F, ORF1a:T3090I, ORF1a:L3201F, ORF1a:T3255I, ORF1a:P3395H, ORF1b:P314L, ORF1b:G662S, ORF1b:S959P, ORF1b:R1315C, ORF1b:I1566V, ORF1b:T2163I, ORF3a:T223I, ORF6:D61L, ORF9b:P10S, ORF9b:D33N, S:T19I, S:**A27S**, S:G142D, S:Q183E, S:V213E, S:G252V, S:G339H, S:R346T, S:L368I, S:S371F, S:S373P, S:S375F, S:T376A, S:D405N, S:R408S, S:K417N, S:N440K, S:V445P, S:G446S, S:N460K, S:S477N, S:T478K, S:E484A, S:F486S, S:F490S, S:Q498R, S:N501Y, S:Y505H, S:D614G, S:H655Y, S:N679K, S:P681H, S:N764K, S:D796Y, S:Q954H |
| 59 | OQ587523 | **XBB** | E:T11A, M:Q19E, M:A63T, N:P13L, ORF1a:K47R, ORF1a:G82D, ORF1a:S135R, ORF1a:T842I, ORF1a:G1307S, ORF1a:L3027F, ORF1a:T3090I, ORF1a:L3201F, ORF1a:T3255I, ORF1a:P3395H, ORF1b:P314L, ORF1b:G662S, ORF1b:S959P, ORF1b:R1315C, ORF1b:I1566V, ORF1b:T2163I, ORF3a:T223I, ORF6:D61L, ORF9b:P10S, S:T19I |
| 60 | OQ569692 | **XBB.3** | E:T11A, M:Q19E, M:A63T, N:P13L, N:R203K, N:G204R, N:S413R, ORF1a:K47R, ORF1a:G82D, ORF1a:S135R, ORF1a:T842I, ORF1a:G1307S, ORF1a:L3027F, ORF1a:T3090I, ORF1a:L3201F, ORF1a:T3255I, ORF1a:P3395H, ORF1b:P314L, ORF1b:G662S, ORF1b:S959P, ORF1b:R1315C, ORF1b:I1566V, ORF1b:I1998V, ORF1b:T2163I, ORF3a:T223I, ORF6:D61L, ORF9b:P10S, S:T19I, S:**A27S**, S:G142D, S:H146Q, S:Q183E, S:V213E, S:G339H, S:R346T, S:L368I, S:S371F, S:S373P, S:S375F, S:T376A, S:D405N, S:R408S, S:K417N, S:N440K, S:V445P, S:G446S,S:N460K, S:S477N, S:T478K, S:E484A, S:F486S, S:F490S, S:Q498R, S:N501Y, S:Y505H, S:D614G, S:H655Y, S:N679K, S:P681H, S:N764K, S:D796Y, S:Q954H |
| 61 | OQ587524 | **XBB.5** | E:T11A, M:Q19E, M:A63T, N:P13L, N:R203K, N:G204R, N:S413R, ORF1a:K47R, ORF1a:G82D, ORF1a:S135R, ORF1a:T842I, ORF1a:V1104A, ORF1a:G1307S, ORF1a:L3027F, ORF1a:T3090I, ORF1a:L3201F, ORF1a:T3255I, ORF1a:P3395H, ORF1b:P314L, ORF1b:G662S, ORF1b:S959P, ORF1b:R1315C, ORF1b:I1566V, ORF1b:T2163I, ORF3a:T223I, ORF6:D61L, ORF9b:P10S, S:T19I |
| 76 | OQ569693 | **XBB.3** | E:T11A,M:Q19E,M:A63T,N:P13L,N:R203K,N:G204R,N:S413R,ORF1a:K47R,ORF1a:G82D, ORF1a:S135R, ORF1a:T842I, ORF1a:G1307S, ORF1a:L3027F, ORF1a:T3090I, ORF1a:L3201F, ORF1a:T3255I, ORF1a:P3395H, ORF1a:L3606F, ORF1b:P314L, ORF1b:G662S, ORF1b:S959P, ORF1b:R1315C, ORF1b:I1566V, ORF1b:I1998V, ORF1b:T2163I, ORF3a:T223I, ORF6:D61L, , ORF9b:P10S, S:T19I, S:**A27S**, S:G142D, S:H146Q, S:Q183E, S:V213E, S:G339H, S:R346T, S:L368I, S:S371F, S:S373P, S:S375F, S:T376A, S:D405N, S:R408S, S:K417N, S:N440K, S:V445P, S:G446S, S:N460K, S:S477N, S:T478K, S:E484A, S:F486S, S:F490S, S:Q498R, S:N501Y, S:Y505H, S:D614G, S:H655Y, S:N679K, S:P681H, S:N764K, S:D796Y, S:Q954H |

| | | | |
|---|---|---|---|
| 77 | OQ569694 | **XBB.5** | E:T11A, M:Q19E, M:A63T, N:P13L, N:R203K, N:G204R, N:S413R, ORF1a:K47R, ORF1a:G82D, ORF1a:S135R, ORF1a:T842I, ORF1a:G1307S, ORF1a:L3027F, ORF1a:T3090I, ORF1a:L3201F, ORF1a:T3255I, ORF1a:P3395H, ORF1b:P314L, ORF1b:G662S, ORF1b:S959P, ORF1b:R1315C, ORF1b:I1566V, ORF1b:T2163I, ORF3a:T223I, ORF6:D61L, ORF9b:P10S, S:T19I, S:**A27S**, S:G142D, S:H146Q, S:Q183E, S:V213E, S:G339H, S:R346T, S:L368I, S:S371F, S:S373P, S:S375F, S:T376A, S:D405N, S:R408S, S:K417N, S:N440K, S:V445P, S:G446S, S:N460K, S:S477N, S:T478K, S:E484A, S:F486S, S:F490S, S:Q498R, S:N501Y, S:Y505H, S:D614G, S:A653V, S:H655Y, S:N679K, S:P681H, S:N764K, S:D796Y, S:Q954H |
| 82 | OQ587525 | **XBB.3** | E:T11A, M:Q19E, M:A63T, N:P13L, N:R203K, N:G204R, N:S413R, ORF1a:K47R, ORF1a:G82D, ORF1a:S135R, ORF1a:T842I, ORF1a:G1307S, ORF1a:L3027F, ORF1a:T3090I, ORF1a:L3201F, ORF1a:T3255I, ORF1a:P3395H, ORF1b:P314L, ORF1b:G662S, ORF1b:S959P, ORF1b:R1315C, ORF1b:I1566V, ORF1b:I1998V, ORF1b:T2163I, ORF3a:T223I, ORF6:D61L, ORF9b:P10S, S:G142D, S:H146Q, S:Q183E, S:V213E, S:G339H, S:R346T, S:L368I, S:S371F, S:S373P, S:S375F, S:T376A, S:D405N, S:R408S, S:K417N, S:N440K, S:V445P, S:G446S, S:N460K, S:S477N, S:T478K, S:E484A, S:F486S, S:F490S, S:Q498R, S:N501Y, S:Y505H, S:D614G, S:H655Y, S:N679K, S:P681H, S:N764K, S:D796Y, S:Q954H |
| 94 | OQ587526 | **XBB.3** | E:T11A, M:Q19E, M:A63T, N:P13L, N:R203K, N:G204R, N:S413R, ORF1a:K47R, ORF1a:G82D, ORF1a:S135R, ORF1a:T842I, ORF1a:G1307S,ORF1a:H2357Y,ORF1a:L3027F, ORF1a:T3090I, ORF1a:L3201F,ORF1a:T3255I,ORF1a:P3395H,ORF1b:P314L, ORF1b:G662S,ORF1b:S959P,ORF1b:R1315C,ORF1b:I1566V,ORF1b:I1998V,ORF1b:T2163I, ORF3a:T223I, ORF6:D61L, ORF9b:P10S, S:T19I |
| 95 | OQ587527 | **XBB.3** | E:T11A, M:A63T, N:P13L, N:R203K, N:G204R, N:S413R,ORF1a:K47R, ORF1a:G82D, ORF1a:S135R, ORF1a:T842I, ORF1a:G1307S, ORF1a:T1881I, ORF1a:G2868S, ORF1a:L3027F, ORF1a:T3090I, ORF1a:L3201F, ORF1a:T3255I, ORF1a:P3395H, ORF1b:P314L, ORF1b:G662S, ORF1b:S959P, ORF1b:R1315C, ORF1b:I1566V, ORF1b:I1998V, ORF1b:T2163I, ORF3a:T223I, ORF6:D61L, ORF9b:P10S, S:T19I, S:**A27S**, S:G339H, S:R346T, S:L368I, S:S371F, S:S373P, S:S375F, S:T376A, S:D405N, S:R408S, S:K417N, S:N440K, S:V445P, S:G446S, S:N460K, S:S477N, S:T478K, S:E484A, S:F486S, S:F490S, S:Q498R, S:N501Y, S:Y505H, S:D614G, S:H655Y, S:N679K, S:P681H, S:N764K, S:D796Y, S:Q954H |
| 111 | OQ587528 | **XBB.3** | E:T11A, M:Q19E, M:A63T, N:P13L, N:R203K, N:G204R, N:S413R, ORF1a:K47R, ORF1a:R73H, ORF1a:G82D, ORF1a:S135R, ORF1a:G728A, ORF1a:G2284Q, ORF1a:P2287H, ORF1b:P314L, ORF9b:P10S, S:T19I, S:**A27S**, S:F92N, S:G142D |
| 124 | OQ587529 | **XBB** | E:T11A, M:Q19E, M:A63T, N:P13L, N:R203K, N:G204R, N:S413R, ORF1a:K47R, ORF1a:G82D, ORF1a:S135R, ORF1a:H374Y, ORF1a:T842I, ORF1a:G1307S, ORF1a:L3027F, ORF1a:T3090I, ORF1a:L3201F, ORF1a:T3255I, ORF1a:P3395H, ORF1b:P314L, ORF1b:G662S, ORF1b:S959P, ORF1b:R1315C, ORF1b:I1566V, ORF1b:T2163I, ORF3a:T223I, ORF6:D61L, ORF9b:P10S, S:T19I |
| 133 | OQ569695 | **XBB.3** | E:T11A, M:Q19E, M:A63T, N:P13L, N:A152V, N:S187L, N:R203K, N:G204R, N:S413R, ORF1a:K47R, ORF1a:G82D, ORF1a:S135R, ORF1a:T842I, ORF1a:G1307S, ORF1a:L3027F, ORF1a:T3090I, ORF1a:L3201F, ORF1a:T3255I, ORF1a:P3395H, ORF1b:P314L, ORF1b:G662S, ORF1b:S959P, ORF1b:R1315C, ORF1b:I1566V, ORF1b:I1998V, ORF1b:T2163I, ORF3a:T223I, ORF6:D61L, ORF9b:P10S, S:T19I, S:**A27S**, S:G142D, S:H146Q, S:Q183E, S:V213E, S:G339H, S:R346T, S:L368I, S:S371F, S:S373P, S:S375F, S:T376A, S:D405N, S:R408S, S:K417N, S:N440K, S:V445P, S:G446S, S:N460K, S:S477N, S:T478K, S:E484A, S:F486S, S:F490S, S:Q498R, S:N501Y, S:Y505H, S:D614G, S:H655Y, S:N679K, S:P681H, S:N764K, S:D796Y, S:Q954H |
| 143 | OQ587530 | **XBB.3** | E:T11A, M:Q19E, M:A63T, N:P13L, N:R203K, N:G204R, N:S413R, ORF1a:K47R, ORF1a:G82D, ORF1a:S135R, ORF1a:T842I, ORF1a:G1307S, ORF1a:L3027F, ORF1a:T3090I, ORF1a:L3201F, ORF1a:T3255I, ORF1a:P3395H, ORF1b:P314L, ORF1b:G662S, ORF1b:S959P, ORF1b:R1315C, ORF1b:I1566V, ORF1b:P1727H, ORF1b:I1998V, ORF1b:T2163I, ORF3a:T223I, ORF6:D61L, ORF9b:P10S, S:T19I |
| 144 | OQ587531 | **XBB.1** | E:T11A, M:Q19E, M:A63T, N:P13L, N:R36Q, N:R203K, N:G204R, N:S413R, ORF1a:K47R, ORF1a:S135R, ORF1a:T842I, ORF1a:G1307S, ORF1a:L3027F, ORF1a:T3090I, ORF1a:L3201F, ORF1a:T3255I, ORF1a:P3395H, ORF1b:P314L, ORF1b:G662S, ORF1b:S959P, ORF1b:R1315C, ORF1b:I1566V, ORF1b:T2163I, ORF3a:T223I, ORF6:D61L, ORF9b:P10S, ORF9b:D33N, S:T19I, S:**A27S**, S:G142D, S:H146Q, S:Q183E, S:V213E, S:G252V, S:G339H, S:R346T, S:L368I, S:S371F, S:S373P, S:S375F, S:T376A, S:D405N, S:R408S, S:K417N, S:N440K, S:V445P, S:G446S, S:N460K, S:S477N, S:T478K, S:E484A, S:F486S, S:F490S, S:Q498R, S:N501Y, S:Y505H, S:D614G, S:H655Y, S:N679K, S:P681H, S:N764K, S:D796Y, S:Q954H |

| | | | |
|---|---|---|---|
| 160 | OQ587532 | **XBB.3** | E:T11A, M:Q19E, M:A63T, N:P13L, N:R203K, N:G204R, N:S413R, ORF1a:K47R, ORF1a:G82D, ORF1a:S135R, ORF1a:T842I, ORF1a:G1307S, ORF1a:L3027F, ORF1a:T3090I, ORF1a:L3201F, ORF1a:T3255I, ORF1a:P3395H, ORF1a:T4174I, ORF1b:P314L, ORF1b:G662S, ORF1b:S959P, ORF1b:R1315C, ORF1b:I1566V, ORF1b:V1760F, ORF1b:I1998V, ORF1b:T2163I, ORF3a:T223I, ORF6:D61L, ORF9b:P10S, S:T19I, S:**A27S**, S:F92I, S:G142D, S:H146Q, S:Q183E, S:V213E, S:G339H, S:R346T, S:L368I, S:S371F, S:S373P, S:S375F, S:T376A, S:D405N, S:R408S, S:K417N, S:N440K, S:V445P, S:G446S, S:N460K, S:S477N, S:T478K, S:E484A, S:F486S, S:F490S, S:Q498R, S:N501Y, S:D614G, S:H655Y, S:N679K, S:P681H, S:N764K, S:D796Y, S:Q954H |
| 161 | OQ587533 | **XBB.3** | E:T11A, M:Q19E, M:A63T, N:P13L, N:R203K, N:G204R, N:S413R, ORF1a:K47R, ORF1a:G82D, ORF1a:S135R, ORF1a:P276T, ORF1a:T842I, ORF1a:G1307S, ORF1a:L3027F, ORF1a:T3090I, ORF1a:L3201F, ORF1a:T3255I, ORF1a:P3395H, ORF1b:P314L, ORF1b:G662S, ORF1b:S959P, ORF1b:R1315C, ORF1b:I1566V, ORF1b:I1998V, ORF1b:T2163I, ORF3a:T223I, ORF6:D61L, ORF9b:P10S, S:T19I |
| 162 | OQ569696 | **XBB.3** | E:T11A, M:Q19E, M:A63T, N:P13L, N:R203K, N:G204R, N:S413R, ORF1a:K47R, ORF1a:G82D, ORF1a:S135R, ORF1a:T842I, ORF1a:G1307S, ORF1a:L3027F, ORF1a:T3090I, ORF1a:L3201F, ORF1a:T3255I, ORF1a:P3395H, ORF1b:P314L, ORF1b:G662S, ORF1b:S959P, ORF1b:T981S, ORF1b:R1315C, ORF1b:I1566V, ORF1b:I1998V, ORF1b:T2163I, ORF3a:T223I, ORF6:D61L, ORF9b:P10S, S:T19I, S:**A27S**, S:G142D, S:H146Q, S:Q183E, S:V213E, S:T323I, S:G339H, S:R346T, S:L368I, S:S371F, S:S373P, S:S375F, S:T376A, S:D405N, S:R408S, S:K417N, S:N440K, S:V445P, S:G446S, S:N460K, S:S477N, S:T478K, S:E484A, S:F486S, S:F490S, S:Q498R, S:N501Y, S:Y505H, S:D614G, S:H655Y, S:N679K, S:P681H, S:N764K, S:D796Y, S:Q954H |
| 163 | OQ587534 | **XBB** | E:T11A, M:Q19E, M:A63T, N:P13L, N:R203K, N:G204R, N:S413R, ORF1a:K47R, ORF1a:G82D, ORF1a:S135R, ORF1a:T842I, ORF1a:G1307S, ORF1a:L3027F, ORF1a:T3090I, ORF1a:L3201F, ORF1a:T3255I, ORF1a:P3395H, ORF1b:P314L, ORF1b:G662S, ORF1b:S959P, ORF1b:R1315C, ORF1b:I1566V, ORF1b:T2163I, ORF3a:T223I, ORF6:D61L, ORF9b:P10S, S:T19I |
| 174 | OQ569697 | **XBB.3** | E:T11A, M:Q19E, M:A63T, N:P13L, N:R203K, N:G204R, N:S413R, ORF1a:K47R, ORF1a:G82D, ORF1a:S135R, ORF1a:T842I, ORF1a:G1307S, ORF1a:L3027F, ORF1a:T3090I, ORF1a:L3201F, ORF1a:T3255I, ORF1a:P3395H, ORF1b:P314L, ORF1b:G662S, ORF1b:S959P, ORF1b:R1315C, ORF1b:I1566V, ORF1b:I1998V, ORF1b:K2063N, ORF1b:T2163I, ORF3a:T223I, ORF6:D61L, ORF9b:P10S, S:T19I, S:**A27S**, S:G142D, S:H146Q, S:Q183E, S:V213E, S:G339H, S:R346T, S:L368I, S:S371F, S:S373P, S:S375F, S:T376A, S:D405N, S:R408S, S:K417N, S:N440K, S:V445P, S:G446S, S:N460K, S:S477N, S:T478K, S:E484A, S:F486S, S:F490S, S:Q498R, S:N501Y, S:Y505H, S:D614G, S:H655Y, S:N679K, S:P681H, S:N764K, S:D796Y, S:Q954H |
| 177 | OQ587535 | **XBB.1** | E:T11A, M:Q19E, M:A63T, N:P13L, N:R203K, N:G204R, N:S413R, ORF1a:K47R, ORF1a:S135R, ORF1a:T842I, ORF1a:G1307S, ORF1a:V2866M, ORF1a:L3027F, ORF1a:L3201F, ORF1a:T3255I, ORF1a:P3395H, ORF1b:P314L, ORF1b:G662S, ORF1b:S959P, ORF1b:R1315C, ORF1b:I1566V, ORF1b:T2163I, ORF3a:D210E, ORF3a:T223I, ORF6:D61L, ORF8:G8*, ORF9b:P10S, S:T19I, S:**A27S**, S:G142D, S:H146Q, S:Q183E, S:V213E, S:G252V, S:G339H, S:L368I, S:S371F, S:S373P, S:S375F, S:T376A, S:D405N, S:R408S, S:K417N, S:N440K, S:V445P, S:G446S, S:N460K |
| 178 | OQ587536 | **XBB.1** | E:T11A, M:Q19E, M:A63T, N:P13L, N:R203K, N:G204R, N:S413R, ORF1a:K47R, ORF1a:M85I, ORF1a:S135R, ORF1a:T842I, ORF1a:G1307S, ORF1a:L3027F, ORF1a:T3090I, ORF1a:L3201F, ORF1a:T3255I, ORF1a:P3395H, ORF1b:P314L, ORF1b:G662S, ORF1b:S959P, ORF1b:R1315C, ORF1b:I1566V, ORF1b:T2163I, ORF3a:T223I, ORF6:D61L, ORF8:G8*, ORF9b:P10S, S:T19I |
| 179 | OQ569698 | **XBB.1** | E:T11A, M:Q19E, M:A63T, N:P13L, N:R203K, N:G204R, N:S413R, ORF1a:K47R, ORF1a:M85I, ORF1a:S135R, ORF1a:T842I, ORF1a:G1307S, ORF1a:L3027F, ORF1a:T3090I, ORF1a:L3201F, ORF1a:T3255I, ORF1a:P3395H, ORF1b:P314L, ORF1b:G662S, ORF1b:S959P, ORF1b:R1315C, ORF1b:I1566V, ORF1b:T2163I, ORF3a:T223I, ORF6:D61L, ORF8:G8*, ORF9b:P10S, S:T19I |
| 180 | OQ587537 | **XBB.1** | E:T11A, M:Q19E, N:P13L, ORF1a:K47R, ORF1a:S135R, ORF1a:T842I, ORF1a:G1307S, ORF1a:G1819S, ORF1a:S1857P, ORF1a:A2123V, ORF1b:P314L, ORF1b:G662S, ORF1b:S959P, ORF1b:R1315C, ORF1b:I1566V, ORF1b:T2163I, ORF3a:T223I, ORF6:D61L, ORF7a:L30H, ORF8:G8*, ORF9b:I5T, ORF9b:P10S, S:T19I, S:**A27S**, S:S94A, S:G142D, S:H146Q, S:Q183E, S:V213E, S:G252V, S:G339H, S:R346T, S:L368I, S:S371F, S:S373P, S:S375F, S:T376A, S:D405N, S:R408S, S:K417N, S:N440K, S:V445P, S:G446S, S:N460K, S:S477N, S:T478K, S:P507T, S:D614G, S:H655Y, S:N679K, S:P681H, S:N764K, S:D796Y, S:Q954H |

| | | | |
|---|---|---|---|
| 181 | OQ587538 | **XBB.3** | E:T11A, M:Q19E, M:A63T, N:P13L, N:R203K, N:G204R, N:S413R, ORF1a:K47R, ORF1a:G82D, ORF1a:S135R, ORF1a:T842I, ORF1a:G1307S, ORF1a:L3027F, ORF1a:T3090I, ORF1a:L3201F, ORF1a:T3255I, ORF1a:P3395H, ORF1b:P314L, ORF1b:G575R, ORF1b:G662S, ORF1b:S959P, ORF1b:R1315C, ORF1b:I1566V, ORF1b:I1998V,ORF1b:T2163I,ORF3a:T223I,ORF6:D61L,ORF9b:P10S,S:T19I |
| 182 | OQ587539 | **XBB** | E:T11A, M:Q19E, M:A63T, N:P13L, ORF1a:K47R, ORF1a:G82D, ORF1a:S135R, ORF1a:T842I, ORF1a:G1307S, ORF1a:L3027F, ORF1a:L3201F, ORF1a:T3255I, ORF1a:P3395H, ORF1b:P314L, ORF3a:T223I, ORF6:D61L, ORF9b:I5T, ORF9b:P10S, S:T19I, S:**A27S**, S:F92L, S:A93S, S:S94T, S:G142D, S:H146Q, S:D614G, S:H655Y, S:N679K, S:P681H, S:N764K, S:D796Y, S:Q954H |
| 183 | OQ652056 | **XBB** | E:T11A, N:P13L, N:M101*, ORF1b:P314L, ORF1b:G662S, ORF1b:S959P, ORF1b:R1315C, ORF1b:I1566V, ORF1b:T2163I, ORF3a:T223I, ORF9b:P10S, ORF9b:K97N, ORF9b:*98R, S:T19I, S:**A27S**, S:R408S, S:K417N, S:N440K, S:V445P |
| 184 | OQ569699 | **XBB.1** | E:T11A, M:Q19E, M:A63T, N:P13L, N:R203K, N:G204R, N:S413R, ORF1a:K47R, ORF1a:S135R, ORF1a:T842I, ORF1a:G1307S, ORF1a:G1819S, ORF1a:L3027F, ORF1a:T3090I, ORF1a:L3201F, ORF1a:T3255I, ORF1a:P3395H, ORF1b:P314L, ORF1b:G662S, ORF1b:S959P, ORF1b:R1315C, ORF1b:I1566V, ORF1b:T2163I, ORF3a:T223I, ORF6:D61L, ORF8:G8*, ORF9b:I5T, ORF9b:P10S, S:T19I, S:**A27S**, S:G142D, S:H146Q, S:Q183E, S:V213E, S:G252V, S:G339H, S:R346T, S:L368I, S:S371F, S:S373P, S:S375F, S:T376A, S:D405N, S:R408S, S:K417N, S:N440K, S:V445P, S:G446S, S:N460K, S:S477N, S:T478K, S:E484A, S:F486S, S:F490S, S:Q498R, S:N501Y, S:Y505H, S:D614G, S:H655Y, S:N679K, S:P681H, S:N764K, S:D796Y, S:Q954H |
| 185 | OQ569700 | **XBB.1.9** | E:T11A, M:Q19E, M:A63T, N:P13L, N:R203K, N:G204R, N:S413R, ORF1a:K47R, ORF1a:S135R, ORF1a:T842I, ORF1a:G1307S, ORF1a:G1819S, ORF1a:Y2141H, ORF1a:L3027F, ORF1a:T3090I, ORF1a:L3201F, ORF1a:T3255I, ORF1a:P3395H, ORF1b:P314L, ORF1b:G662S, ORF1b:S959P, ORF1b:R1315C, ORF1b:I1566V, ORF1b:T2163I, ORF3a:T223I, ORF6:D61L, ORF8:G8*, ORF9b:I5T, ORF9b:P10S, S:T19I, S:**A27S**, S:G142D, S:H146Q, S:Q183E, S:V213E, S:G252V, S:G339H, S:R346T, S:L368I, S:S371F, S:S373P, S:S375F, S:T376A, S:D405N, S:R408S, S:K417N, S:N440K, S:V445P, S:G446S, S:N460K, S:S477N, S:T478K, S:E484A, S:F486S, S:F490S, S:Q498R, S:N501Y, S:Y505H, S:D614G, S:H655Y, S:N679K, S:P681H, S:N764K, S:D796Y,,S:Q954H |
| 186 | OQ569701 | **XBB.1.9** | E:T11A, M:Q19E, M:A63T, N:P13L, N:R203K, N:G204R, N:S413R, ORF1a:K47R, ORF1a:S135R, ORF1a:T842I, ORF1a:G1307S, ORF1a:G1819S, ORF1a:Y2141H, ORF1a:L3027F, ORF1a:T3090I, ORF1a:L3201F, ORF1a:T3255I, ORF1a:P3395H, ORF1b:P314L, ORF1b:G662S, ORF1b:S959P, ORF1b:R1315C, ORF1b:I1566V, ORF1b:T2163I, ORF3a:T223I, ORF6:D61L, ORF8:G8*, ORF9b:I5T, ORF9b:P10S, S:T19I, S:**A27S**, S:G142D, S:H146Q, S:Q183E, S:V213E, S:G252V, S:G339H, S:R346T, S:L368I, S:S371F, S:S373P, S:S375F, S:T376A, S:D405N, S:R408S, S:K417N, S:N440K, S:V445P, S:G446S, S:N460K, S:S477N, S:T478K, S:E484A, S:F486S, S:F490S, S:Q498R, S:N501Y, S:Y505H, S:D614G, S:H655Y, S:N679K, S:P681H, S:N764K, S:D796Y, S:Q954H |
| 187 | OQ569702 | **XBB.1.9** | E:T11A, M:Q19E, M:A63T, N:P13L, N:R203K, N:G204R, N:G238S, N:S413R, ORF1a:K47R, ORF1a:S135R, ORF1a:T842I, ORF1a:G1307S, ORF1a:G1819S, ORF1a:Y2141H, ORF1a:L3027F, ORF1a:T3090I, ORF1a:L3201F, ORF1a:T3255I, ORF1a:P3395H, ORF1b:P314L, ORF1b:G662S, ORF1b:S959P, ORF1b:R1315C, ORF1b:I1566V, ORF1b:T2163I, ORF3a:T223I, ORF6:D61L, ORF8:G8*, ORF9b:I5T, ORF9b:P10S, S:T19I, S:**A27S**, S:G142D, S:H146Q, S:Q183E, S:V213E, S:G252V, S:G339H, S:R346T, S:L368I, S:S371F, S:S373P, S:S375F, S:T376A, S:D405N, S:R408S, S:K417N, S:N440K, S:V445P, S:G446S, S:N460K, S:S477N, S:T478K, S:E484A, S:F486S, S:F490S, S:Q498R, S:N501Y, S:Y505H, S:D614G, S:H655Y, S:N679K, S:P681H, S:N764K, S:D796Y, S:Q954H |
| 188 | OQ587540 | **XBB.1** | E:T11A, M:Q19E, M:A63T, N:P13L, ORF1a:K47R, ORF1a:S135R, ORF1a:T842I, ORF1a:G1307S, ORF1b:P314L, ORF1b:G662S, ORF1b:S959P, ORF1b:R1315C, ORF1b:I1566V, ORF1b:T2163I, ORF3a:K198N, ORF3a:T223I, ORF6:D61L, ORF8:G8*, ORF9b:P10S, ORF9b:E65D, S:T19I |
| 189 | OQ569703 | **XBB.1** | E:T11A, M:Q19E, M:A63T, N:P13L, N:R203K, N:G204R, N:S413R, ORF1a:K47R, ORF1a:S135R, ORF1a:T842I, ORF1a:G1307S, ORF1a:G1819S, ORF1a:L3027F, ORF1a:T3090I, ORF1a:L3201F, ORF1a:T3255I, ORF1a:P3395H, ORF1b:P314L, ORF1b:G662S, ORF1b:S959P, ORF1b:R1315C, ORF1b:I1566V, ORF1b:T2163I, ORF3a:T223I, ORF6:D61L, ORF8:G8*, ORF9b:I5T, ORF9b:P10S, S:T19I, S:**A27S**, S:G142D, S:H146Q, S:Q183E, S:V213E, S:G252V, S:G339H, S:R346T, S:L368I, S:S371F, S:S373P, S:S375F, S:T376A, S:D405N, S:R408S, S:K417N, S:N440K, S:V445P, S:G446S, S:N460K, S:S477N, S:T478K, S:E484A, S:F486S, S:F490S, S:Q498R, S:N501Y, S:D614G, S:H655Y, S:N679K, S:P681H, S:N764K, S:D796Y, S:Q954H |

| | | | |
|---|---|---|---|
| 190 | OQ587541 | **XBB.2** | E:T11A, M:Q19E, M:A63T, M:I73V, N:P13L, N:R203K, N:G204R, N:S413R, ORF1a:K47R, ORF1a:S135R, ORF1a:T842I, ORF1a:G1307S, ORF1a:T1637I, ORF1a:L3027F, ORF1a:T3090I, ORF1a:L3201F, ORF1a:T3255I, ORF1a:P3395H, ORF1b:P314L, ORF1b:G662S, ORF1b:S959P, ORF1b:R1315C, ORF1b:I1566V, ORF1b:T2163I, ORF3a:T223I, ORF6:D61L, ORF8:S54P, ORF9b:P10S, S:T19I, S:**A27S**, S:G142D, S:H146Q, S:Q183E, S:V213E, S:D253G, S:G339H, S:R346T, S:L368I, S:S371F, S:S373P, S:S375F, S:T376A, S:D405N, S:R408S, S:K417N, S:N440K, S:V445P, S:G446S, S:N460K, S:S477N, S:T478K, S:E484A, S:F486S, S:F490S, S:Q498R, S:N501Y, S:D614G, S:H655Y, S:N679K, S:P681H, S:N764K, S:D796Y, S:Q954H |
| 191 | OQ569704 | **XBB.1** | E:T11A, M:Q19E, M:A63T, N:P13L, N:R36Q, N:R203K.N:G204R, N:S413R, ORF1a:K47R, ORF1a:H81R, ORF1a:S135R, ORF1a:T842I, ORF1a:G1307S, ORF1a:K1720R, ORF1a:T1881I, ORF1a:L3027F, ORF1a:T3090I, ORF1a:L3201F, ORF1a:T3255I, ORF1a:P3395H, ORF1b:P314L, ORF1b:G662S, ORF1b:S959P, ORF1b:R1315C, ORF1b:I1566V, ORF1b:T2163I, ORF3a:T223I, ORF6:D61L, ORF9b:P10S, ORF9b:D33N, S:T19I, S:**A27S**, S:D88K, S:G89W, S:V90G, S:G142D, S:H146Q, S:Q183E, S:V213E, S:G252V, S:G339H, S:R346T, S:L368I, S:S371F, S:S373P, S:S375F, S:T376A, S:D405N, S:R408S, S:K417N, S:N440K, S:V445P, S:G446S, S:N460K, S:S477N, S:T478K, S:E484A, S:F486S, S:F490S, S:Q498R, S:N501Y, S:D614G, S:A647S, S:H655Y, S:N679K, S:P681H, S:N764K, S:D796Y, S:Q954H, S:N969K |
| 192 | OQ652057 | **XBB.1** | E:T11A, M:Q19E, N:P13L, N:R203K, N:G204R, N:S413R, ORF1a:K47R, ORF1a:S135R, ORF1a:T842I, ORF1a:G1307S, ORF1a:L3027F, ORF1a:T3090I, ORF1a:L3201F, ORF1a:T3255I, ORF1a:P3395H, ORF1b:P314L, ORF1b:G662S, ORF1b:S959P, ORF1b:R1315C, ORF1b:I1566V, ORF1b:T2163I, ORF3a:T223I, ORF6:D61L, ORF8:G8*, ORF9b:P10S, ORF9b:T60I, S:T19I, S:F43R |
| 193 | OQ569705 | **XBB.2** | E:T11A, M:Q19E, M:A63T, N:P13L, N:R203K, N:G204R, N:S413R, ORF1a:K47R, ORF1a:S135R, ORF1a:T842I, ORF1a:G1307S, ORF1a:L3027F, ORF1a:T3090I, ORF1a:L3201F, ORF1a:T3255I, ORF1a:P3395H, ORF1a:I3779V, ORF1b:P314L, ORF1b:G662S, ORF1b:S959P, ORF1b:R1315C, ORF1b:I1566V, ORF1b:T2163I, ORF3a:T223I, ORF6:D61L, ORF9b:P10S, S:T19I, S:**A27S**, S:A67S, S:G142D, S:H146Q, S:Q183E, S:V213E, S:D253G, S:G339H, S:R346T, S:L368I, S:S371F, S:S373P, S:S375F, S:T376A, S:D405N, S:R408S, S:K417N, S:N440K, S:V445P, S:G446S, S:N460K, S:S477N, S:T478K, S:E484A, S:F486S, S:F490S, S:Q498R, S:N501Y, S:P521Q, S:D614G, S:H655Y, S:N679K, S:P681H, S:N764K, S:D796Y, S:Q954H |
| 194 | OQ569706 | **XBB.1** | E:T11A, M:Q19E, M:A63T, N:P13L, N:R203K, N:G204R, N:S413R, ORF1a:K47R, ORF1a:S135R, ORF1a:A540V, ORF1a:T842I, ORF1a:G1307S, ORF1a:L3027F, ORF1a:T3090I, ORF1a:L3201F, ORF1a:T3255I, ORF1a:P3395H, ORF1b:P314L, ORF1b:G662S, ORF1b:S959P, ORF1b:R1315C, ORF1b:I1566V, ORF1b:T2163I, ORF3a:T223I, ORF6:D61L, ORF8:G8*, ORF9b:P10S, S:T19I, S:**A27S**, S:G142D, S:H146Q, S:Q183E, S:V213E, S:G252V, S:G339H, S:R346T, S:L368I, S:S371F, S:S373P, S:S375F, S:T376A, S:D405N, S:R408S, S:K417N, S:N440K, S:V445P, S:G446S, S:N460K, S:S477N, S:T478K, S:E484A, S:F486S, S:F490S, S:Q498R, S:N501Y, S:D614G, S:H655Y, S:N679K, S:P681H, S:N764K, S:D796Y, S:Q954H, S:N969K |
| 195 | OQ587542 | **XBB.1** | E:T11A, M:Q19E, M:A63T, N:P13L, N:R203K, N:G204R, N:A252S, N:P368L, N:S413R, ORF1a:K47R, ORF1a:R99H, ORF1a:S135R, ORF1a:F651L, ORF1a:T842I, ORF1a:L1110F, ORF1a:G1307S, ORF1a:A2379V, ORF1a:L3027F, ORF1a:T3090I, ORF1a:L3201F, ORF1a:T3255I, ORF1a:P3395H, ORF1b:P314L, ORF1b:G662S, ORF1b:S959P, ORF1b:R1315C, ORF1b:I1566V, ORF1b:T2163I, ORF3a:T24I, ORF3a:T223I, ORF6:D61L, ORF9b:P10S, S:T19I |
| 196 | OQ652149 | **XBB.3** | E:T11A, M:Q19E, M:A63T, N:P13L, N:R203K, N:G204R, N:S413R, ORF1a:K47R, ORF1a:G82D, ORF1a:S135R, ORF1a:T842I, ORF1a:G1307S, ORF1a:L3027F, ORF1a:T3090I, ORF1a:L3201F, ORF1a:T3255I, ORF1a:P3395H, ORF1b:P314L, ORF1b:G662S, ORF1b:S959P, ORF1b:R1315C, ORF1b:I1566V, ORF1b:I1998V, ORF1b:T2163I, ORF3a:T223I, ORF6:D61L, ORF9b:P10S, S:T19I, S:**A27S**, S:F86I, S:G142D, S:H146Q, S:Q183E, S:V213E, S:G339H, S:R346T, S:L368I, S:S371F, S:S373P, S:S375F, S:T376A, S:D405N, S:R408S, S:K417N, S:N440K, S:V445P, S:G446S, S:N460K, S:S477N, S:T478K, S:E484A, S:F486S, S:F490S, S:Q498R, S:D614G, S:H655Y, S:N679K, S:P681H, S:N764K, S:D796Y, S:Q954H |

| | | | |
|---|---|---|---|
| 197 | OQ587543 | **XBB.3** | E:T11A, M:Q19E, M:A63T, N:P13L, N:R203K, N:G204R, N:S413R, ORF1a:K47R, ORF1a:G82D, ORF1a:S135R, ORF1a:T842I, ORF1a:G1307S, ORF1a:G2545S, ORF1a:L3027F, ORF1a:T3090I, ORF1a:L3201F, ORF1a:T3255I, ORF1a:P3395H, ORF1b:P314L, ORF1b:G662S, ORF1b:S959P, ORF1b:R1315C, ORF1b:I1566V, ORF1b:I1998V, ORF1b:T2163I, ORF3a:T223I, ORF6:D61L, ORF9b:P10S, S:T19I |
| 198 | OQ569707 | **XBB.3** | E:T11A, M:Q19E, M:A63T, N:P13L, N:R203K, N:G204R, N:S413R, ORF1a:K47R, ORF1a:G82D, ORF1a:S135R, ORF1a:T842I, ORF1a:G1307S, ORF1a:L3027F, ORF1a:T3090I, ORF1a:L3201F, ORF1a:T3255I, ORF1a:P3395H, ORF1b:P314L, ORF1b:G662S, ORF1b:S959P, ORF1b:R1315C, ORF1b:I1566V, ORF1b:I1998V, ORF1b:T2163I, ORF3a:T223I, ORF6:D61L, ORF9b:P10S, S:T19I, S:**A27S**, S:G142D, S:H146Q, S:Q183E, S:V213E, S:G339H, S:R346T, S:L368I, S:S371F, S:S373P, S:S375F, S:T376A, S:D405N, S:R408S, S:K417N, S:N440K, S:V445P, S:G446S, S:N460K, S:S477N, S:T478K, S:E484A, S:F486S, S:F490S, S:Q498R, S:N501Y, S:D614G, S:H655Y, S:N679K, S:P681H, S:N764K, S:D796Y, S:Q954H |
| 199 | OQ587544 | **XBB.1** | E:T11A, M:Q19E, M:A63T, N:P13L, N:R203K, N:G204R, N:S413R, ORF1a:K47R, ORF1a:S135R, ORF1a:D144Y, ORF1a:T842I, ORF1a:G1307S, ORF1a:L3027F, ORF1a:T3090I, ORF1a:L3201F, ORF1a:T3255I, ORF1a:P3395H, ORF1b:P314L, ORF1b:G662S, ORF1b:S959P, ORF1b:R1315C, ORF1b:I1566V, ORF1b:T2163I, ORF3a:T223I, ORF6:D61L, ORF9b:P10S, S:T19I, S:**A27S**, S:D614G, S:H655Y, S:N679K, S:P681H, S:N764K, S:D796Y, S:Q954H |
| 200 | OQ587545 | **XBB.2** | E:T11A, M:Q19E, M:A63T, N:P13L, ORF1a:K47R, ORF1a:S135R, ORF1a:T842I, ORF1a:G1307S, ORF1a:A2909S, ORF1a:L3027F, ORF1a:T3090I, ORF1a:L3201F, ORF1a:T3255I, ORF1a:P3395H, ORF1b:P314L, ORF1b:G662S, ORF1b:S959P, ORF1b:R1315C, ORF1b:I1566V, ORF1b:T2163I, ORF3a:T24I, ORF3a:D155Y, ORF3a:T223I, ORF6:D61L, ORF9b:P10S, S:T19I |
| 201 | OQ587546 | **XBB.3** | E:T11A, M:Q19E, M:A63T, N:P13L, N:A90S, N:R203K, N:G204R, N:S413R, ORF1a:R24H, ORF1a:K47R, ORF1a:G82D, ORF1a:S135R, ORF1a:T842I, ORF1a:G1307S, ORF1a:S2911T, ORF1a:L3027F, ORF1a:T3090I, ORF1a:L3201F, ORF1a:T3255I, ORF1a:P3395H, ORF1a:T4355I, ORF1b:P314L, ORF1b:G662S, ORF1b:S959P, ORF1b:R1315C, ORF1b:I1566V, ORF1b:I1998V, ORF1b:T2163I, ORF3a:T223I, ORF6:D61L, ORF9b:P10S, ORF9b:E86D, S:T19I |
| 202 | OQ587547 | **XBB.1** | E:T11A, M:Q19E, M:A63T, N:P13L, N:R203K, N:G204R, N:S413R, ORF1a:K47R, ORF1a:S135R, ORF1a:T842I, ORF1a:G1307S, ORF1a:G1819S, ORF1a:L3027F, ORF1a:L3201F, ORF1a:T3255I, ORF1a:P3395H, ORF1a:T4175I, ORF1b:P314L, ORF1b:G662S, ORF1b:S959P, ORF1b:R1315C, ORF1b:I1566V, ORF1b:T2163I, ORF3a:T223I, ORF6:D61L, ORF8:G8*, ORF9b:I5T, ORF9b:P10S, S:T19I, S:**A27S**, S:D614G, S:H655Y, S:N679K, S:P681H, S:N764K, S:D796Y, S:Q954H |
| 203 | OQ587548 | **XBB.3** | E:T11A, M:Q19E, M:A63T, N:P13L, N:R203K, N:G204R, N:S413R, ORF1a:K47R, ORF1a:G82D, ORF1a:S135R, ORF1a:T842I, ORF1a:G1307S, ORF1a:L3027F, ORF1a:T3090I, ORF1a:L3201F, ORF1a:T3255I, ORF1a:P3395H, ORF1b:P314L, ORF1b:G662S, ORF1b:S959P, ORF1b:R1315C, ORF1b:I1566V, ORF1b:T2163I, ORF3a:T223I, ORF6:D61L, ORF9b:P10S, S:T19I, S:**A27S**, S:G142D, S:H146Q, S:Q183E, S:V213E, S:G339H, S:R346T, S:L368I, S:S371F, S:S373P, S:S375F, S:T376A, S:D405N, S:R408S, S:K417N, S:N440K, S:V445P, S:G446S, S:N460K, S:S477N, S:T478K, S:E484A, S:F486S |
| 204 | OQ569708 | **XBB.3** | E:T11A, M:Q19E, M:A63T, N:P13L, N:R203K, N:G204R, N:S413R, ORF1a:K47R, ORF1a:G82D, ORF1a:S135R, ORF1a:T842I, ORF1a:G1307S, ORF1a:S1952L, ORF1a:L3027F, ORF1a:T3090I, ORF1a:L3201F, ORF1a:T3255I, ORF1a:P3395H, ORF1b:P314L, ORF1b:G662S, ORF1b:S959P, ORF1b:R1315C, ORF1b:I1566V, ORF1b:I1998V, ORF1b:T2163I, ORF3a:T223I, ORF6:D61L, ORF9b:P10S, S:T19I, S:**A27S**, S:F86L, S:G142D, S:H146Q, S:Q183E, S:V213E, S:G339H, S:R346T, S:L368I, S:S371F, S:S373P, S:S375F, S:T376A, S:D405N, S:R408S, S:K417N, S:N440K, S:V445P, S:G446S, S:N460K, S:S477N, S:T478K, S:E484A, S:F486S, S:F490S, S:Q498R, S:N501Y, S:D614G, S:H655Y, S:N679K, S:P681H, S:N764K, S:D796Y, S:Q954H |
| 205 | OQ587549 | **XBB.5** | E:T11A, M:Q19E, M:A63T, N:P13L, N:R203K, N:G204R, N:S413R, ORF1a:K47R, ORF1a:G82D, ORF1a:S135R, ORF1a:R550C, ORF1a:T842I, ORF1a:G1307S, ORF1a:L3027F, ORF1a:T3090I, ORF1a:L3201F, ORF1a:T3255I, ORF1a:P3395H, ORF1b:P314L, ORF1b:G662S, ORF1b:S959P, ORF1b:R1315C, ORF1b:I1566V, ORF1b:T2163I, ORF3a:T223I, ORF6:D61L, ORF9b:P10S, S:T19I |
| 206 | OQ587550 | **XBB.5** | E:T11A, M:Q19E, M:A63T, N:P13L, N:R203K, N:G204R, N:S413R, ORF1a:K47R, ORF1a:G82D, ORF1a:S135R, ORF1a:T842I, ORF1a:G1307S, ORF1a:L3027F, ORF1a:T3090I, ORF1a:L3201F, ORF1a:T3255I, ORF1a:P3395H, ORF1b:P314L, ORF1b:G662S, ORF1b:S959P, ORF1b:R1315C, |

| | | | |
|---|---|---|---|
| | | | ORF1b:I1566V, ORF1b:T2163I, ORF3a:T223I, ORF6:D61L, ORF9b:P10S, S:T19I, S:**A27S**, S:G142D, S:H146Q, S:Q183E, S:V213E, S:G339H, S:R346T, S:L368I, S:S371F, S:S373P, S:S375F, S:T376A, S:D405N, S:R408S, S:K417N, S:N440K, S:V445P, S:G446S, S:N460K |
| 207 | OQ587551 | **XBB.3** | E:T11A, M:Q19, M:A63T, N:P13L, N:R203K, N:G204R, N:S413R, ORF1a:K47R, ORF1a:G82D, ORF1a:S135R, ORF1a:T842I, ORF1a:G1307S, ORF1a:L3027F, ORF1a:T3090I, ORF1a:L3201F, ORF1a:T3255I, ORF1a:P3395H, ORF1b:P314L, ORF1b:G662S, ORF1b:S959P, ORF1b:R1315C, ORF1b:I1566V, ORF1b:I1998V, ORF1b:T2163I, ORF3a:T223I, ORF6:D61L, ORF9b:P10S, S:T19I, S:**A27S** |
| 208 | OQ587552 | **XBB.1.5** | E:T11A, M:Q19E, M:A63T, N:P13L, N:R203K, N:G204R, N:S413R, ORF1a:K47R, ORF1a:S135R, ORF1a:T842I, ORF1a:G1307S, ORF1a:L3027F, ORF1a:L3201F, ORF1a:T3255I, ORF1a:P3395H, ORF1b:P314L, ORF1b:G662S, ORF1b:S959P, ORF1b:R1315C, ORF1b:I1566V, ORF1b:T2163I, ORF3a:T223I, ORF6:D61L, ORF8:G8*, ORF9b:P10S, S:T19I, S:**A27S**, S:D614G, S:H655Y, S:N679K, S:P681H, S:N764K, S:D796Y, S:Q954H |
| 209 | OQ587553 | **XBB.1.5** | E:T11A, M:Q19E, M:A63T, N:P13L, N:R203K, N:G204R, N:S413R, ORF1a:K47R, ORF1a:S135R, ORF1a:T842I, ORF1a:G1307S, ORF1a:L3027F, ORF1a:T3090I, ORF1a:L3201F, ORF1a:T3255I, ORF1a:P3395H, ORF1b:P314L, ORF1b:G662S, ORF1b:S959P, ORF1b:R1315C, ORF1b:I1566V, ORF1b:T2163I, ORF3a:T223I, ORF6:D61L, ORF8:G8*, ORF9b:P10S, S:T19I |
| 210 | OQ652067 | **XBB.1** | E:T11A, M:Q19E, M:A63T, N:P13L, N:R203K, N:G204R, N:S413R, ORF1a:K47R, ORF1a:S135R, ORF1a:G353C, ORF1a:T842I, ORF1a:G1307S, ORF1a:L3027F, ORF1a:E3073K, ORF1a:C3093V, ORF1a:T3095N, ORF1a:L3829F, ORF1b:P314L, ORF1b:G662S, ORF1b:S959P, ORF1b:R1315C, ORF1b:I1566V, ORF1b:D1746Y, ORF1b:T2163I, ORF3a:T223I, ORF6:D61L, ORF8:G8*, ORF9b:P10S, ORF9b:N55S, S:T19I, S:**A27S**, S:G142D, S:H146Q, S:Q183E, S:V213E, S:G252V, S:G339H, S:L368I, S:S371F, S:S373P, S:S375F, S:T376A, S:D405N, S:R408S, S:K417N, S:N440K, S:V445P, S:G446S, S:N460K, S:T478K, S:Q498R, S:N501Y, S:Y505H, S:D614G, S:H655Y, S:N679K, S:P681H, S:N764K, S:D796Y, S:Q954H |
| 211 | OQ569709 | **XBB.1.5** | E:T11A, M:Q19E, M:A63T, N:P13L, N:R203K, N:G204R, N:S413R, ORF1a:K47R, ORF1a:S135R, ORF1a:T842I, ORF1a:G1307S, ORF1a:L3027F, ORF1a:T3090I, ORF1a:L3201F, ORF1a:T3255I, ORF1a:P3395H, ORF1b:P314L, ORF1b:G662S, ORF1b:S959P, ORF1b:R1315C, ORF1b:I1566V, ORF1b:T2163I, ORF3a:T223I, ORF6:D61L, ORF8:G8*, ORF9b:P10S, S:T19I, S:**A27S**, S:G142D, S:H146Q, S:Q183E, S:V213E, S:G252V, S:G339H, S:F347C, S:L368I, S:S371F, S:S373P, S:S375F, S:T376A, S:D405N, S:R408S, S:K417N, S:N440K, S:V445P, S:G446S, S:N460K, S:T478K, S:E484A, S:F486P, S:F490S, S:Q498, S:N501Y, S:D614G, S:H655Y, S:N679K, S:P681H, S:N764K, S:D796Y, S:Q954H |
| 212 | OQ587554 | **XBB.1** | E:T11A, M:Q19E, M:A63T, N:P13L, N:R203K, N:G204R, N:S413R, ORF1a:K47R, ORF1a:S135R, ORF1a:T842I, ORF1a:G1307S, ORF1a:L3027F, ORF1a:T3090I, ORF1a:L3201F, ORF1a:T3255I, ORF1a:P3371S, ORF1a:P3395H, ORF1b:P314L, ORF1b:G662S, ORF1b:A869V, ORF1b:S959P, ORF1b:R1315C, ORF1b:I1566V, ORF1b:T2163I, ORF3a:T223I, ORF6:D61L, ORF8:G8*, ORF9b:P10S, S:T19I |
| 213 | OQ587555 | **XBB.1.5** | E:T11A, M:Q19E, M:A63T, N:P13L, N:R203K, N:G204R, N:S413R, ORF1a:K47R, ORF1a:S135R, ORF1a:T842I, ORF1a:G1307S, ORF1a:L3027F, ORF1a:T3090I, ORF1a:L3201F, ORF1a:T3255I, ORF1a:P3395H, ORF1b:P314L, ORF1b:G662S, ORF1b:S959P, ORF1b:R1315C, ORF1b:I1566V, ORF1b:T2163I, ORF3a:T223I, ORF6:D61L, ORF8:G8*, ORF9b:P10S, S:T19I, S:**A27S**, S:N87D, S:G142D, S:H146Q, S:Q183E, S:V213E, S:G252V, S:G339H, S:R346T, S:L368I, S:S371F, S:S373P, S:S375F, S:T376A, S:D405N, S:R408S, S:K417N, S:N440K, S:V445P, S:G446S, S:N460K, S:S477N, S:T478K, S:E484A, S:F486P, S:F490S, S:Q498R, S:N501Y, S:D614G, S:H655Y, S:N679K, S:P681H, S:N764K, S:D796Y, S:Q954H, S:N969K |
| 214 | OQ587556 | **XBB** | E:T11A, M:Q19E, M:A63T, N:P13L, N:R203K, N:G204R, N:S413R, ORF1a:K47R, ORF1a:G82D, ORF1a:S135R, ORF1a:T842I, ORF1a:G1307S, ORF1a:L3027F, ORF1a:T3090I, ORF1a:L3201F, ORF1a:T3255I, ORF1a:P3395H, ORF1b:P314L, ORF1b:G662S, ORF1b:S959P, ORF1b:R1315C, ORF1b:I1566V, ORF1b:T2163I, ORF3a:T223I, ORF6:D61L, ORF9b:P10S, S:T19I, S:**A27S**, S:G142D, S:H146Q, S:Q183E, S:V213E, S:G339H, S:R346T, S:L368I, S:S371F, S:S373P, S:S375F, S:T376A, S:D405N, S:R408S, S:K417N, S:N440K, S:V445P, S:G446S, S:N460K, S:S477N, S:T478K, S:E484A, S:F486S |
| 215 | OQ587557 | **XBB.1** | E:T11A, M:Q19E, M:A63T, N:P13L, N:R203K, N:G204R, N:S413R, ORF1a:K47R, ORF1a:S135R, ORF1a:T842I, ORF1a:G1307S, ORF1a:L3027F, ORF1a:L3201F, ORF1a:T3255I, ORF1a:P3395H, ORF1b:P314L, ORF1b:G662S, ORF1b:S959P, ORF1b:R1315C, ORF1b:I1566V, ORF1b:T2163I, ORF3a:T223I, ORF6:D61L, ORF8:G8*, ORF9b:P10S, S:T19I, S:**A27S**, S:G142D, S:H146K, S:Q183E, S:V213E, S:G252V, S:G339H, S:R346T, S:L368I, S:S371F, S:S373P, S:S375F, S:T376A, S:D405N, S:R408S, S:K417N, S:N440K, S:V445P, S:G446S |

| | | | |
|---|---|---|---|
| 216 | OQ652150 | **XBB.1** | E:T11A, M:Q19E, M:A63T, N:P13L, N:R203K, N:G204R, N:S413R, ORF1a:K47R, ORF1a:S135R, ORF1a:T842I, ORF1a:L1130F, ORF1a:G1307S, ORF1a:L3027F, ORF1a:T3090I, ORF1a:L3201F, ORF1a:T3255I, ORF1a:P3395H, ORF1b:P314L, ORF1b:G662S, ORF1b:S959P, ORF1b:R1315C, ORF1b:I1566V, ORF1b:T2163I, ORF3a:T223I, ORF6:D61L, ORF9b:P10S, S:T19I, S:**A27S**, S:G142D, S:H146Q, S:Q183E, S:V213E, S:G252V, S:G339H, S:R346T, S:L368I, S:S371F, S:S373P, S:S375F, S:T376A, S:D405N, S:R408S, S:K417N, S:N440K, S:V445P, S:G446S, S:N460K, S:S477N, S:T478K, S:E484A, S:F486S, S:F490S, S:Q498R, S:N501Y, S:D614G, S:H655Y, S:N679K, S:P681H, S:N764K, S:D796Y, S:Q954H |
| 217 | OQ587558 | **XBB.3** | E:T11A, M:Q19E, M:A63T, N:P13L, N:R203K, N:G204R, N:S413R, ORF1a:K47R, ORF1a:G82D, ORF1a:S135R, ORF1a:T842I, ORF1a:G1307S, ORF1a:D1440G, ORF1a:K1529N, ORF1a:L3027F, ORF1a:T3090I, ORF1a:L3201F, ORF1a:T3255I, ORF1a:P3395H, ORF1b:T250I, ORF1b:P314L, ORF1b:G662S, ORF1b:S959P, ORF1b:R1315C, ORF1b:I1566V, ORF1b:I1998V, ORF1b:T2163I, ORF3a:T223I, ORF6:D61L, ORF9b:P10S, S:T19I |
| 218 | OQ652151 | **XBB.2** | E:T11A, M:Q19E, M:A63T, N:P13L, N:R203K, N:G204R, N:S413R, ORF1a:K47R, ORF1a:S135R, ORF1a:T842I, ORF1a:G1307S, ORF1a:L3027F, ORF1a:T3090I, ORF1a:L3201F, ORF1a:T3255I, ORF1a:P3395H, ORF1a:I3779V, ORF1b:P314L, ORF1b:G662S, ORF1b:S959P, ORF1b:R1315C, ORF1b:I1566V, ORF1b:T2163I, ORF3a:T223I, ORF6:D61L, ORF9b:P10S, S:**A27S**, S:A67S, S:G142D, S:H146Q, S:Q183E, S:V213E, S:D253G, S:G339H, S:R346T, S:L368I, S:S371F, S:S373P, S:S375F, S:T376A, S:D405N, S:R408S, S:K417N, S:N440K, S:V445P, S:G446S, S:N460K,S:S477N, S:T478K, S:E484A, S:F486S, S:F490S, S:Q498R, S:N501Y, S:P521Q, S:D614G, S:H655Y, S:N679K, S:P681H, S:N764K, S:D796Y, S:Q954H |
| 219 | OQ652069 | **XBB.1.5** | E:T11A, M:Q19E, N:P13L, ORF1a:K47R, ORF1a:S135R, ORF1a:T842I, ORF1a:G1307S, ORF1a:L3027F, ORF1b:P314L, ORF3a:L73P, ORF3a:T223I, ORF6:D61L, ORF8:G8*, ORF9b:P10S, S:T19I, S:**A27S**, S:G339H, S:L368I, S:S371F, S:S373P, S:S375F, S:T376A, S:D405N, S:R408S, S:K417N, S:N440K, S:V445P, S:G446S |
| 220 | OQ652070 | **XBB.2** | N:R203K, N:G204R, ORF1a:K47R, ORF1a:S135R, ORF1a:D203N, ORF1a:Y799I, ORF1a:T842I, ORF1a:G1307S, ORF1a:L3201F, ORF1a:T3255, ORF1a:P3395H, ORF1b:P314L, ORF3a:T223I, ORF6:D61L, ORF9b:P10S, ORF9b:E65G, S:T19I, S:G142D, S:H146Q, S:Q183E, S:V213E, S:D253G |
| 221 | OQ569710 | **XBB.2** | E:T11A, M:Q19E, M:A63T, N:P13L, N:R203K, N:G204R, N:S413R, ORF1a:K47R, ORF1a:S135R, ORF1a:T842I, ORF1a:G1307S, ORF1a:L3027F, ORF1a:T3090I, ORF1a:L3201F, ORF1a:T3255I, ORF1a:P3395H, ORF1b:P314L, ORF1b:G662S, ORF1b:S959P, ORF1b:R1315C, ORF1b:I1566V, ORF1b:T2163I, ORF1b:A2222V, ORF3a:T223I, ORF6:D61L, ORF9b:P10S, S:T19I, S:**A27S**, S:G142D, S:H146P, S:Q183E, S:V213E, S:D253G, S:G339H, S:R346T, S:L368I, S:S371F, S:S373P, S:S375F, S:T376A, S:D405N, S:R408S, S:K417N, S:N440K, S:V445P, S:G446S, S:N460K, S:S477N, S:T478K, S:E484A, S:F486S, S:F490S, S:Q498R, S:N501Y, S:Y505H, S:Q613H, S:D614G, S:H655Y, S:N679K, S:P681H, S:N764K, S:D796Y, S:Q954H |
| 222 | OQ587559 | **XBB.1.2** | E:T11A, M:Q19E, M:A63T, N:P13L, N:R203K, N:G204R, N:S413R, ORF1a:K47R, ORF1a:S135R, ORF1a:T842I, ORF1a:G950S, ORF1a:G1307S, ORF1a:L3027F, ORF1a:T3090I, ORF1a:L3201F, ORF1a:T3255I, ORF1a:P3395H, ORF1b:P314L, ORF1b:G662S, ORF1b:S959P, ORF1b:T1050N, ORF1b:R1315C, ORF1b:I1566V, ORF1b:T2163I, ORF3a:T223I, ORF6:D61L, ORF8:G8*, ORF9b:P10S, S:T19I |
| 223 | OQ587560 | **XBB.2** | E:T11A, M:Q19E, M:A63T, N:P13L, N:R203K, N:G204R, N:S413R, ORF1a:K47R, ORF1a:S135R, ORF1a:T842I, ORF1a:G1307S, ORF1a:P1977L, ORF1a:L3027F, ORF1a:T3090I, ORF1a:L3201F, ORF1a:T3255I, ORF1a:P3395H, ORF1b:P314L, ORF1b:G662S, ORF1b:S959P, ORF1b:R1315, ORF1b:I1566V, ORF1b:T2163I, ORF3a:T223I, ORF6:D61L, ORF9b:P10S, S:T19I, S:**A27S**, S:A67S, S:G142D, S:H146Q, S:Q183E, S:V213E, S:D253G, S:G339H, S:R346T, S:L368I, S:S371F, S:S373P, S:S375F, S:T376A, S:D405N, S:R408S, S:K417, S:N440, S:V445P, S:G446S, S:N460K, S:S477N, S:T478K, S:E484A, S:F486S, S:F490S, S:Q498R, S:N501Y, S:P521Q, S:D614G, S:H655Y, S:N679K, S:P681H, S:N764K, S:D796Y, S:Q954H |
| 224 | OQ652152 | **XBB.1.2** | E:T11A, M:Q19E, M:A63T, N:P13L, N:R203K, N:G204R, N:S413R, ORF1a:K47R, ORF1a:S135R, ORF1a:T842I, ORF1a:G1307S, ORF1a:T1678N, ORF1a:L3027F, ORF1a:T3090I, ORF1a:L3201F, ORF1a:T3255I, ORF1a:P3395H, ORF1b:P314L, ORF1b:G662S, ORF1b:S959P, ORF1b:R1315C, ORF1b:I1566V, ORF1b:Y1648C, ORF1b:T2163I, ORF3a:T223I, ORF6:D61L, ORF8:G8*, ORF9b:P10S, ORF9b:A22V, S:T19I, S:**A27S**, S:G142D, S:H146Q, S:Q183E, S:V213E, S:G252V, S:G339H, S:R346T, S:L368I, S:S371F, S:S373P, S:S375F, S:T376A, S:D405N, S:R408S, S:K417N, S:N440K, S:V445P, S:G446S, S:N460K, S:S477N, S:T478K, S:E484A, S:F486S, S:F490S, S:Q498R, S:N501Y, S:D614G, S:H655Y, S:N679K, S:P681H, S:N764K, S:D796Y, S:Q954H |
| 225 | OQ569711 | **XBB.1.2** | E:T11A, M:Q19E, M:A63T, N:P13L, N:R203K, N:G204R, N:S413R, ORF1a:K47R, ORF1a:S135R, ORF1a:T842I, ORF1a:G1307S, ORF1a:T1678N, ORF1a:L3027F, ORF1a:T3090I, ORF1a:L3201F, ORF1a:T3255I, ORF1a:P3395H, ORF1b:P314L, ORF1b:G662S, ORF1b:S959P, ORF1b:R1315C, ORF1b:I1566V, ORF1b:Y1648C, ORF1b:T2163I, ORF3a:T223I, ORF6:D61L, ORF8:G8*, ORF9b:P10S, ORF9b:A22V, S:T19I, S:**A27S**, S:G142D, |

| | | | |
|---|---|---|---|
| | | | S:H146Q, S:Q183E, S:V213E, S:G252V, S:G339H, S:R346T, S:L368I, S:S371F, S:S373P, S:S375F, S:T376A, S:D405N, S:R408S, S:K417N, S:N440K,S:V445P, S:G446S, S:N460K, S:S477N, S:T478K, S:E484A, S:F486S, S:F490S, S:Q498R, S:N501Y, S:D614G, S:H655Y, S:N679K, S:P681H, S:N764K, S:D796Y, S:Q954H |
| 226 | OQ587561 | **XBB.1** | E:T11A, M:Q19E, M:A63T, N:P13L, N:R203K, N:G204R, N:S413R, ORF1a:K47R, ORF1a:S135R, ORF1a:T842I, ORF1a:G1307S, ORF1a:G1705S, ORF1a:T2087I, ORF1a:L3027F, ORF1a:L3201F, ORF1a:T3255I, ORF1a:P3395H, ORF1a:A4357V, ORF1b:P314L, ORF1b:G662S, ORF1b:S959P, ORF1b:R1315C, ORF1b:I1566V, ORF1b:T2163I, ORF3a:T223I, ORF6:D61L, ORF8:G8*, ORF9b:P10S, S:T19I |
| 227 | OQ587562 | **XBB.1** | E:T11A, M:Q19E, M:A63T, N:P13L, N:R203K, N:G204R, N:S413R, ORF1a:K47R, ORF1a:S135R, ORF1a:T842I, ORF1a:G1307S, ORF1a:L3027F, ORF1a:T3090I, ORF1a:L3201F, ORF1a:T3255I, ORF1a:P3395H, ORF1b:P314L, ORF1b:G662S, ORF1b:S959P, ORF1b:R1315C, ORF1b:I1566V, ORF1b:K1828Q, ORF1b:T2163I, ORF3a:T223I, ORF6:D61L, ORF8:G8*, ORF9b:P10S, S:T19I, S:**A27S**, S:G142D, S:H146Q, S:Q183E, S:V213E, S:G252V, S:G339H, S:R346T, S:L368I, S:S371F, S:S373P, S:S375F, S:T376A, S:D405N, S:R408S, S:K417N, S:N440K, S:V445P, S:G446S, S:N460K, S:S477N, S:T478K, S:E484A, S:F486S, S:F490S, S:Q498R, S:N501Y, S:Y505H, S:D614G, S:H655Y, S:N679K, S:P681H, SN764K, S:D796Y, S:Q954H |
| 228 | OQ587563 | **XBB.1** | E:T11A, M:Q19E, M:A63T, N:P13L, N:R203K, N:G204R, N:S413R, ORF1a:K47R, ORF1a:S135R, ORF1a:T842I, ORF1a:G1307S, ORF1a:P2287S, ORF1a:L3027F, ORF1a:T3090I, ORF1a:L3201F, ORF1a:T3255I, ORF1a:P3395H, ORF1b:P314L, ORF1b:G662S, ORF1b:S959P, ORF1b:R1315C, ORF1b:I1566, ORF1b:T2163I, ORF3a:T223I, ORF6:D61L, ORF7a:A8V, ORF9b:P10S, S:T19I |
| 229 | OQ587564 | **XBB.1** | E:T11A, M:Q19E, M:A63T, N:P13L, N:R203K, N:G204R, N:S413R, ORF1a:K47R, ORF1a:P80L, ORF1a:S135R, ORF1a:T842I, ORF1a:G1307S, ORF1a:L3027F, ORF1a:T3090I, ORF1a:L3201F, ORF1a:T3255I, ORF1a:P3395H, ORF1b:P314L, ORF1b:G662S, ORF1b:S959P, ORF1b:R1315C, ORF1b:I1566V, ORF1b:T2163I, ORF3a:T223I, ORF6:D61L, ORF8:G8*, ORF9b:P10S, S:T19I, S:**A27S** |
| 230 | OQ587565 | **XBB** | E:T11A, M:Q19E, N:P13L, N:G129S, N:R203K, N:G204R, N:S413R, ORF1a:K47R, ORF1a:G82D, ORF1a:S135R, ORF1a:T842I, ORF1a:G1307S, ORF1a:V1915I, ORF1a:L2367I, ORF1a:L3027F, ORF1a:T3090I, ORF1a:L3201F, ORF1a:T3255I, ORF1a:P3395H, ORF1b:P314L, ORF1b:G662S, ORF1b:S959P, ORF1b:E1264D, ORF1b:R1315C, ORF1b:I1566V, ORF1b:T2163I, ORF3a:T223I, ORF6:D61L, ORF9b:P10S, S:T19I, S:**A27S**, S:S31F, S:V62L, S:G142D, S:H146Q, S:Q183E, S:V213E, S:G339H, S:R346T, S:L368I, S:S371F, S:S373P, S:S375F, S:T376A, S:D405N, S:R408S, S:K417N, S:N440K, S:V445P, S:G446S, S:N460K, S:S477N, S:T478K, S:E484A, S:F486P, S:F490S, S:Q498R, S:N501Y, S:D614G, S:H655Y, S:N679K, S:P681H, S:N764K, S:D796Y, S:Q954H |
| 231 | OQ587566 | **XBB.1.9.1** | E:T11A, M:Q19E, M:A63T, N:P13L, N:R203K, N:G204R, N:S413R, ORF1a:K47R, ORF1a:S135R, ORF1a:C657Y, ORF1a:T842I, ORF1a:G1307S, ORF1a:G1819S, ORF1a:L3027F, ORF1a:T3090I, ORF1a:L3201F, ORF1a:T3255I, ORF1a:P3395H, ORF1a:T4175I, ORF1b:P314L, ORF1b:G662S, ORF1b:S959P, ORF1b:R1315C, ORF1b:I1566V, ORF1b:T2163I, ORF3a:T223I, ORF6:D61L, ORF8:G8*, ORF9b:I5T, ORF9b:P10S, S:T19I |
| 232 | OQ569712 | **XBB.2** | E:T11A, M:Q19E, M:A63T, N:P13L, N:R203K, N:G204R, N:S413R, ORF1a:K47R, ORF1a:S135R, ORF1a:T842I, ORF1a:G1307S, ORF1a:G2091S, ORF1a:L3027F, ORF1a:T3090I, ORF1a:L3201F, ORF1a:T3255I, ORF1a:P3395H, ORF1b:P314L, ORF1b:G662S, ORF1b:R1315C, ORF1b:I1566V, ORF1b:T2163I, ORF3a:T223I, ORF6:D61L, ORF7a:A13V, ORF9b:P10S, S:T19I, S:**A27S**, S:G142D, S:H146Q, S:Q183E, S:V213E, S:D253G, S:G339H, S:R346T, S:L368I, S:S371F, S:S373P, S:S375F, S:T376A, S:D405N, S:R408S, S:K417N, S:N440K, S:V445P, S:G446S, S:N460K, S:S477N, S:T478K, S:E484A, S:F486P, S:F490S, S:Q498R, S:N501Y, S:Y505H, S:P521S, S:D614G, S:H655Y, S:N679K, S:P681H, S:N764K, S:D796Y, S:Q954H |
| 233 | OQ587567 | **XBB.2** | E:T11A, M:Q19E, M:A63T, N:P13L, N:A50V, N:R203K, N:G204R, N:S413R, ORF1a:K47R, ORF1a:S135R, ORF1a:T842I, ORF1a:G1307S, ORF1a:L3027F, ORF1a:T3090I, ORF1a:L3201F, ORF1a:T3255I, ORF1a:P3395H, ORF1b:P314L, ORF1b:G662S, ORF1b:S959P, ORF1b:R1315C, ORF1b:I1566V, ORF1b:T2163I, ORF3a:T223I, ORF6:D61L, ORF9b:P10S, ORF9b:R47C, S:T19I, S:**A27S** |
| 234 | OQ587568 | **XBB.1.9** | E:T11A, M:Q19E, M:A63T, N:P13L, N:R203K, N:G204R, N:S413R, ORF1a:E36D, ORF1a:K47R, ORF1a:S135R, ORF1a:T842I, ORF1a:N1262D, ORF1a:G1307S, ORF1a:G1819S, ORF1a:P2134S, ORF1a:L3027F, ORF1a:L3201F, ORF1a:T3255I, ORF1a:P3395H, ORF1a:T4175I, ORF1b:P314L, ORF1b:G662S, ORF1b:S959P, ORF1b:R1315C, ORF1b:I1322V, ORF1b:S1408A, ORF1b:I1566V, ORF1b:T2163I, ORF3a:T223I, ORF6:D61L, ORF8:G8*, ORF9b:I5T, ORF9b:P10S, S:T19I, S:**A27S**, S:D614G, S:H655Y, S:N679K, S:P681H, S:N764K, S:D796Y, S:Q954H |
| 235 | OQ569713 | **XBB.1** | E:T11A, M:Q19E, M:A63T, N:P13L, N:R203K, N:G204R, N:S413R, ORF1a:K47R, ORF1a:S135R, ORF1a:T842I, ORF1a:G1307S, ORF1a:L3027F, ORF1a:T3090I, ORF1a:L3201F, ORF1a:T3255I, ORF1a:P3371S, ORF1a:P3395H, ORF1b:P314L, ORF1b:G662S, ORF1b:S959P, ORF1b:R1315C, |

| | | | |
|---|---|---|---|
| | | | ORF1b:I1566V, ORF1b:T2163I, ORF3a:T223I, ORF6:D61L, ORF8:G8*, ORF9b:P10S, S:T19I, S:**A27S**, S:G142D, S:H146Q, S:Q183E, S:V213E, S:G252V, S:G339H, S:R346T, S:L368I, S:S371F, S:S373P, S:S375F, S:T376A, S:D405N, S:R408S, S:K417N, S:N440K, S:V445P, S:G446S, S:N460K, S:S477N, S:T478K, S:E484A, S:F486S, S:F490S, S:Q498R, S:N501Y, S:Y505H, S:D614G, S:H655Y, S:N679K, S:P681H, S:N764K, S:D796Y, S:Q954H |
| 236 | OQ652153 | **XBB.1.2** | E:T11A, M:Q19E, M:A63T, N:P13L, N:R203K, N:G204R, N:S413R, ORF1a:K47R, ORF1a:S135R, ORF1a:T842I, ORF1a:G1307S, ORF1a:T1678N, ORF1a:L3027F, ORF1a:T3090I, ORF1a:L3201F, ORF1a:T3255I, ORF1a:P3395H, ORF1b:P314L, ORF1b:G662S, ORF1b:S959P, ORF1b:R1315C, ORF1b:I1566V, ORF1b:Y1648C, ORF1b:T2163I, ORF3a:T223I, ORF6:D61L, ORF8:G8*, ORF9b:P10S, ORF9b:A22V, S:T19I, S:**A27S**, S:G142D, S:H146Q, S:Q183E, S:V213E, S:G252V, S:G339H, S:R346T, S:L368I, S:S371F, S:S373P, S:S375F, S:T376A, S:D405N, SR408S, S:K417N, S:N440K, S:V445P, S:G446S, S:N460K, S:S477N, S:T478K, S:E484A, S:F486S, S:F490S, S:Q498R, S:N501Y, S:D614G, S:H655Y, S:N679K, S:P681H, S:N764K, S:D796Y, S:Q954H |
| 237 | OQ569714 | **XBB.1.5** | E:T11A, M:Q19E, M:A63T, N:P13L, N:R203K, N:G204R, N:S413R, ORF1a:K47R, ORF1a:S135R, ORF1a:T842I, ORF1a:G1307S, ORF1a:T2300I, ORF1a:L3027F, ORF1a:T3090I, ORF1a:L3201F, ORF1a:T3255I, ORF1a:P3395H, ORF1b:P218L, ORF1b:P314L, ORF1b:G662S, ORF1b:S959P, ORF1b:R1315C, ORF1b:I1566V, ORF1b:T2163I, ORF3a:T223I, ORF6:D61L, ORF8:G8*, ORF9b:P10S, S:T19I, S:**A27S**, S:G142D, S:H146Q, S:Q183E, S:V213E, S:G252V, S:G339H, S:R346T, S:L368I, S:S371F, S:S373P, S:S375F, S:T376A, S:D405N, S:R408S, S:K417N, S:N440K, S:V445P, S:G446S, S:N460K, S:S477N, S:T478K, S:E484A, S:F486P, S:F490S, S:Q498R, S:N501Y, S:Y505H, S:D614G, S:H655Y, S:N679K, S:P681H, S:N764K, S:D796Y, S:Q954H |
| 238 | OQ587569 | **XBB.1.5** | E:T11A, M:Q19E, M:A63T, N:P13L, N:R203K, N:G204R, N:S413R, ORF1a:K47R, ORF1a:S135R, ORF1a:T842I, ORF1a:G1307S, ORF1a:T2300I, ORF1a:L3027F, ORF1a:T3090, ORF1a:L3201F, ORF1a:T3255, ORF1a:P3395H, ORF1b:P218L, ORF1b:P314L, ORF1b:G662S, ORF1b:S959P, ORF1b:R1315C, ORF1b:I1566V, ORF1b:T2163I, ORF3a:T223I, ORF6:D61L, ORF8:G8*, ORF9b:P10S, S:T19I, S:**A27S** |
| 239 | OQ569715 | **XBB.1** | E:T11A, M:Q19E, M:A63T, N:P13L, N:R203K, N:G204R, N:S413R, ORF1a:K47R, ORF1a:S135R, ORF1a:T842I, ORF1a:G1307S, ORF1a:L3027F, ORF1a:T3090I, ORF1a:L3201F, ORF1a:T3255I, ORF1a:P3395H, ORF1b:P314L, ORF1b:G662S, ORF1b:S959P, ORF1b:R1315C, ORF1b:I1566V, ORF1b:T2163I, ORF3a:W45R, ORF3a:T223I, ORF6:D61L, ORF8:G8*, ORF9b:P10S, S:T19I, S:**A27S**, S:G142D, S:H146Q, S:Q183E, S:V213E, S:G252V, S:G339H, S:R346T, S:L368I, S:S371F, S:S373P, S:S375F, S:T376A, S:D405N, S:R408S, S:K417N, S:N440K, S:V445P, S:G446S,S:N460K, S:S477N, S:T478K, S:E484A, S:F486S, S:F490S, S:Q498R, S:N501Y, S:D614G, S:H655Y, S:N679K, S:P681H, S:T747I, S:N764K, S:D796Y, S:Q954H |
| 240 | OQ587570 | **XBB.1** | E:T11A, M:Q19E, N:P13L, N:S78N, N:R203K, N:G204R, N:S413R, ORF1a:K47R, ORF1a:L92F, ORF1a:S135R, ORF1a:T842I, ORF1a:G1307S, ORF1a:P2287S, ORF1a:L3027F, ORF1a:T3090I, ORF1a:L3201F, ORF1a:T3255I, ORF1a:P3395H, ORF1b:P314L, ORF1b:G662S, ORF1b:S959P, ORF1b:R1315C, ORF1b:I1566V, ORF1b:T2163I, ORF3a:T223I, ORF6:D61L, ORF9b:P10S, ORF9b:A75T, S:T19I |
| 241 | OQ587571 | **XBB.1.9** | E:T11A, M:Q19E, M:A63T, N:P13L, N:R203K, N:G204R, N:T362IN:S413R, ORF1a:K47R, ORF1a:S135R, ORF1a:T842I, ORF1a:P971L, ORF1a:G1307S, ORF1a:G1819S, ORF1a:L3027F, ORF1a:L3201F, ORF1a:T3255I, ORF1a:P3395H, ORF1a:T4175I, ORF1b:P314L, ORF1b:G662S, ORF1b:S959P, ORF1b:R1315C, ORF1b:Q1546H, ORF1b:I1566V, ORF1b:T2163I, ORF3a:T223I, ORF6:D61L, ORF8:G8*, ORF9b:I5T, ORF9b:P10S, S:T19I, S:**A27S**, S:K77T, S:G142D, S:H146Q, S:Q183E, S:V213E, S:G252V, S:R346T, S:L368I, S:S371F, S:S373P, S:S375F, S:T376A, S:D405N, S:R408S, S:K417N, S:N440K, S:V445P, S:G446S, S:N460K |
| 242 | OQ587572 | **XBB.1** | E:T11A, M:Q19E, M:A63T, N:P13L, N:R203K, N:G204R, N:S413R, ORF1a:K47R, ORF1a:P80L, ORF1a:S135R, ORF1a:T842I, ORF1a:G1307S, ORF1a:L3027F, ORF1a:T3090I, ORF1a:L3201F, ORF1a:T3255I, ORF1a:P3395H, ORF1b:P314L, ORF1b:G662S, ORF1b:S959P, ORF1b:R1315C, ORF1b:I1566V, ORF1b:T2163I, ORF3a:T223I, ORF6:D61L, ORF8:G8*, ORF9b:P10S, S:T19I, S:**A27S**, S:G142D, S:H146Q, S:Q183E, S:V213E, S:G252V, S:G339H, S:R346T, S:L368I, S:S371F, S:S373P, S:S375F, S:T376A, S:D405N, S:R408S, S:K417N, S:N440K, S:V445P, S:G446S, S:N460K, S:S477N, S:T478K, S:E484A, S:F486S, S:F490S, S:Q498R, S:N501Y, S:D614G, S:H655Y, S:N679K, S:P681H, S:N764K, S:D796Y, S:Q954H |

| | | | |
|---|---|---|---|
| 243 | OQ587573 | **XBB.1.5** | E:T11A, N:P13L, N:R203K, N:G204RN:S413R, ORF1a:K47R, ORF1a:S135R, ORF1a:T842I, ORF1a:G1307S, ORF1a:I2790T, ORF1a:L3027F, ORF1a:T3090I, ORF1a:L3201F, ORF1a:T3255I, ORF1a:P3395H, ORF1b:P314L, ORF1b:G662S, ORF1b:S959P, ORF1b:R1315C, ORF1b:I1566V, ORF1b:T2163I, ORF3a:I118T, ORF3a:T223I, ORF6:D61L, ORF8:G8*, ORF9b:P10S, S:T19I |
| 244 | OQ587574 | **XBB.1.9.1** | E:T11A, M:Q19E, M:A63T, N:P13L, N:R203K, N:G204R, N:T362IN:S413R, ORF1a:K47R, ORF1a:S135R, ORF1a:T842I, ORF1a:P971L, ORF1a:G1307S, ORF1a:G1819S, ORF1b:P314L, ORF1b:G662S, ORF1b:S959P, ORF1b:R1315C, ORF1b:Q1546H, ORF1b:I1566V, ORF1b:T2163I, ORF3a:T223I, ORF6:D61L, ORF8:G8*, ORF9b:I5T, ORF9b:P10S, S:T19I, S:**A27S**, S:Y91H, S:G142D, S:H146Q, S:Q183E, S:V213E, S:G252V, S:G339H, S:L368I, S:S371F, S:S373P, S:S375F, S:T376A, S:D405N, S:R408S, S:K417N, S:N440K, S:V445P, S:G446S, S:N460K, S:S477N, S:T478K, S:E484A, S:F486P, S:F490S, S:Q498R, S:N501Y, S:Y505H, S:D614G, S:H655Y, S:N679K, S:P681H, S:N764K, S:D796Y, S:Q954H |

**Supplementary Table 7:** Univariate binary regression analysis

| **A) Asymptomatic** | | |
|---|---|---|
| **Characteristics** | **Odds ratio (95% CI)** | **p value** |
| Age (5) | 0.986 (0.910 – 1.067) | 0.720 |
| Gender, male | 1.58 (0.816 – 3.044) | 0.176 |
| Ct value | 0.102 (0.939 – 1.152) | 0.453 |
| History of COVID-19 | 0.586 (0.231 – 1.489) | 0.261 |
| Vaccination (Yes) | 1.280 (0.531 – 3.088) | 0.582 |
| AZD1222 | 1.143 (0.457 – 2.858) | 0.775 |
| BBV152 | 1.475 (0.496 – 4.384) | 0.485 |
| BNT162b/mRNA-1273 | 2.388 (0.495 – 11.52) | 0.278 |
| No. of doses | 1.041 (0.753 – 1.441) | 0.806 |
| Vaccination prior to COVID-19 | 1.534 (0.606 – 3.886) | 0.367 |
| Underlying conditions (Yes) | 0.728 (0.373 – 1.420) | 0.352 |
| Metabolic syndrome | 1.390 (0.547 – 3.536) | 0.489 |
| Pregnancy | 0.429 (0.163 – 1.124) | 0.085 |
| No. of underlying conditions | 1.135 (0.750 – 1.720) | 0.549 |
| XBB.1 | 2.348 (0.141 – 39.171) | 0.552 |
| XBB.2 | 0.783 (0.046 – 13.391) | 0.866 |
| XBB.3 | 1.971 (0.196 – 19.776) | 0.564 |
| XBB.5 | 7.645 (0 – 22.345) | 0.999 |
| **B) Mild** | | |
| **Characteristics** | | |
| Age (5) | 0.964 (0.906 – 1.025) | 0.244 |
| Gender, male | 0.815 (0.490 – 1.356) | 0.431 |
| Ct value | 0.971 (0.896 – 1.053) | 0.477 |
| History of COVID-19 | 0.566 (0.29 – 1.104) | 0.095 |
| Vaccination (Yes) | 3.211 (1.640 – 6.286) | 0.001*** |
| AZD1222 | 3.021 (1.507 – 6.055) | 0.002** |
| BBV152 | 4.327 (1.777 – 10.534) | 0.001*** |
| BNT162b/mRNA-1273 | 1.875 (0.472 – 7.454) | 0.372 |

| | | |
|---|---|---|
| No. of doses | 1.512 (1.165 – 1.962) | 0.002** |
| Vaccination prior to COVID-19 | 3.062 (1.559 – 6.011) | 0.001*** |
| Underlying conditions (Yes) | 0.807 (0.472 – 1.380) | 0.433 |
| Metabolic syndrome | 0.813 (0.411 – 1.609) | 0.553 |
| Pregnancy | 0.764 (0.308 – 1.895) | 0.561 |
| No. of underlying conditions | 1.024 (0.731 – 1.435) | 0.889 |
| XBB.1 | 0.333 (0.124 – 0.898) | 0.030* |
| XBB.2 | 0.733 (0.219 – 2.454) | 0.615 |
| XBB.3 | 1.253 (0.510 – 3.081) | 0.623 |
| XBB.5 | 0.150 (0.014 – 1.560) | 0.112 |
| **C) Moderate/Severe** | | |
| **Characteristics** | | |
| Age (5) | 1.121 (1.029 – 1.220) | 0.009** |
| Gender, male | 0.779 (0.387 – 1.468) | 0.484 |
| Ct value | 1.047 (0.939 – 1.166) | 0.409 |
| History of COVID-19 | 3.634 (1.691 – 7.807) | 0.001*** |
| Vaccination (Yes) | 4.889 (1.133 – 21.101) | 0.033* |
| AZD1222 | 5.946 (1.363 – 25.946) | 0.018* |
| BBV152 | 2.316 (0.402 – 13.353) | 0.348 |
| BNT162b/mRNA-1273 | 5.500 (0.674 – 44.903) | 0.112 |
| No. of doses | 1.541 (1.041 – 2.281) | 0.031* |
| Vaccination prior to COVID-19 | 0.725 (0.156 – 3.358) | 0.681 |
| Underlying conditions (Yes) | 0.996 (0.804 – 1.234) | 0.973 |
| Metabolic syndrome | 1.931 (0.855 – 4.360) | 0.113 |
| Pregnancy | 4.223 (0.551 – 32.377) | 0.166 |
| No. of underlying conditions | 1.380 (0.910 –2.092) | 0.130 |
| XBB.1 | 1.087 (0.304 – 3.881) | 0.898 |
| XBB.2 | 1.250 (0.270 – 5.795) | 0.776 |
| XBB.3 | 0.750 (0.230 – 2.446) | 0.633 |
| XBB.5 | 3.333 (0.414 – 26.857) | 0.258 |
| **D) Hospitalization** | | |
| **Characteristics** | | |

| | | |
|---|---|---|
| Age (5) | 0.923 (0.862 – 0.989) | 0.024* |
| Gender, male | 0.628 (0.355 – 1.112) | 0.111 |
| Ct value | 1.109 (1.008 – 1.221) | 0.035* |
| History of COVID-19 | 0.950 (0.468 – 1.932) | 0.888 |
| Vaccination (Yes) | 1.567 (1.035 – 2.373) | 0.034* |
| AZD1222 | 1.333 (0.557 – 3.193) | 0.519 |
| BBV152 | 2.200 (0.756 – 6.402) | 0.519 |
| BNT162b/mRNA-1273 | 2.667 (0.466 – 15.252) | 0.270 |
| No. of doses | 1.201 (0.873 – 1.653) | 0.260 |
| Vaccination prior to COVID-19 | 1.695 (0.712 – 4.032) | 0.233 |
| Underlying conditions (Yes) | 1.668 (0.768 – 1.930) | 0.005** |
| Metabolic syndrome | 2.449 (1.230 – 4.876) | 0.011* |
| Pregnancy | 3.032 (1.241 – 7.408) | 0.015* |
| No. of underlying conditions | 0.436 (0.299 – 0.636) | <0.0001**** |
| XBB.1 | 1.021 (0.334 – 3.117) | 0.971 |
| XBB.2 | 0.875 (0.233 – 3.284) | 0.843 |
| XBB.3 | 1.072 (0.410 – 2.803) | 0.877 |
| XBB.5 | 0 (0 – 0) | 0.999 |
| **E) O2 support** | | |
| **Characteristics** | | |
| Age (5) | 0.750 (0.555 – 1.013) | 0.061 |
| Gender, male | 0.888 (0.123 – 6.417) | 0.906 |
| Ct value | 1.312 (0.890 – 1.933) | 0.170 |
| History of COVID-19 | 0.733 (0.074 – 7.221) | 0.790 |
| Vaccination (Yes) | 3.081 (0.566 – 16.781) | 0.193 |
| AZD1222 | 12.174 (1.060 – 139.759) | 0.045* |
| BBV152 | 3.652 (0.314 – 42.480) | 0.301 |
| BNT162b/mRNA-1273 | 14. 156 (0 – 24.371) | 0.999 |
| No. of doses | 1.777 (0.687 – 4.596) | 0.236 |
| Vaccination prior to COVID-19 | 8.955 (1.201 – 66.755) | 0.032* |
| Underlying conditions (Yes) | 1.276 (0.523 – 3.115) | 0.593 |
| Metabolic syndrome | 4.475 (0.612 – 32.728) | 0.140 |

| | | |
|---|---|---|
| Pregnancy | 0 (0 – 0) | 0.998 |
| No. of underlying conditions | 0.542 (0.196 – 1.496) | 0.237 |
| XBB.1 | 1.773 (0.106 – 29.760) | 0.691 |
| XBB.2 | 0.773 (0.045 – 13.268) | 0.859 |
| XBB.3 | 6.227 (0.376 – 103.241) | 0.202 |
| XBB.5 | 73.651 (0 – 133.478) | 0.999 |
| **F) HDU/ICU** | | |
| **Characteristics** | | |
| Age (5) | 0.747 (0.582 – 0.958) | 0.021* |
| Gender, male | 0.435 (0.078 – 2.427) | 0.343 |
| Ct value | 1.143 (0.860 – 1.519) | 0.356 |
| History of COVID-19 | 0.233 (0.045 – 1.196) | 0.081 |
| Vaccination (Yes) | 4.217 (0.732 – 24.307) | 0.107 |
| AZD1222 | 2.978 (0.516 – 17.206) | 0.223 |
| BBV152 | 14.164 (0 – 25.163) | 0.998 |
| BNT162b/mRNA-1273 | 14.289 (0 – 31.144) | 0.999 |
| No. of doses | 1.519 (0.675 – 3.416) | 0.313 |
| Vaccination prior to COVID-19 | 4.432 (0.767 – 25.597) | 0.096 |
| Underlying conditions (Yes) | 0.933 (0.606 – 1.438) | 0.755 |
| Metabolic syndrome | 4.564 (0.888 – 23.467) | 0.069 |
| Pregnancy | 0 (0 – 0) | 0.998 |
| No. of underlying conditions | 0.517 (0.139 – 0.925) | 0.006** |
| XBB.1 | 1.773 (0.106 – 29.760) | 0.691 |
| XBB.2 | 7.660 (0 – 18.344) | 0.998 |
| XBB.3 | 3.091 (0.269 – 35.545) | 0.365 |
| XBB.5 | 0.045 (0.015 – 0.750) | 0.131 |
| **G) Outcome: Terminal illness** | | |
| **Characteristics** | | |
| Age (5) | 0.509 (0.313 – 0.828) | 0.007** |
| Gender, male | 1.839 (0.330 – 10.231) | 0.487 |
| Ct value | 1.272 (0.929 – 1.741) | 0.134 |
| History of COVID-19 | 0.480 (0.085 – 2.709) | 0.406 |

| | | |
|---|---|---|
| Vaccination (Yes) | 2.600 (0.707 – 9.560) | 0.150 |
| AZD1222 | 0 (0 – 0) | 0.999 |
| BBV152 | 0 (0 – 0) | 0.999 |
| BNT162b/mRNA-1273 | 0 (0 – 0) | 0.999 |
| No. of doses | 1.826 (0.877 – 3.801) | 0.107 |
| Vaccination prior to COVID-19 | 2.267 (0.402 – 12.780) | 0.353 |
| Underlying conditions (Yes) | 1.085 (0.195 – 6.049) | 0.926 |
| Metabolic syndrome | 0.961 (0.109 – 8.444) | 0.971 |
| Pregnancy | 0 (0 – 0) | 0.998 |
| No. of underlying conditions | 0.937 (0.315 – 2.792) | 0.907 |
| XBB.1 | 0 (0 – 0) | 0.999 |
| XBB.2 | 0 (0 – 0) | 0.999 |
| XBB.3 | 0 (0 – 0) | 0.999 |
| XBB.5 | 0 (0 – 0) | 0.999 |

**Supplemental Table 8:** COVID-19 cases reported in Tamil Nadu during September 2022 – January 2023

| Month | No. of samples tested | No. of positives | Positivity (%) |
|---|---|---|---|
| September 2022 | 491883 | 15212 | 3.1 |
| October 2022 | 319863 | 5073 | 1.6 |
| November 2022 | 230271 | 1207 | 0.5 |
| December 2022 | 146438 | 285 | 0.2 |
| January 2023 | 142585 | 202 | 0.1 |
| **Grand total** | **1331040** | **21979** | **1.7** |

**Supplemental Figure 1:** Emergence of XBB variant in Tamil Nadu during September 2022 – January 2023

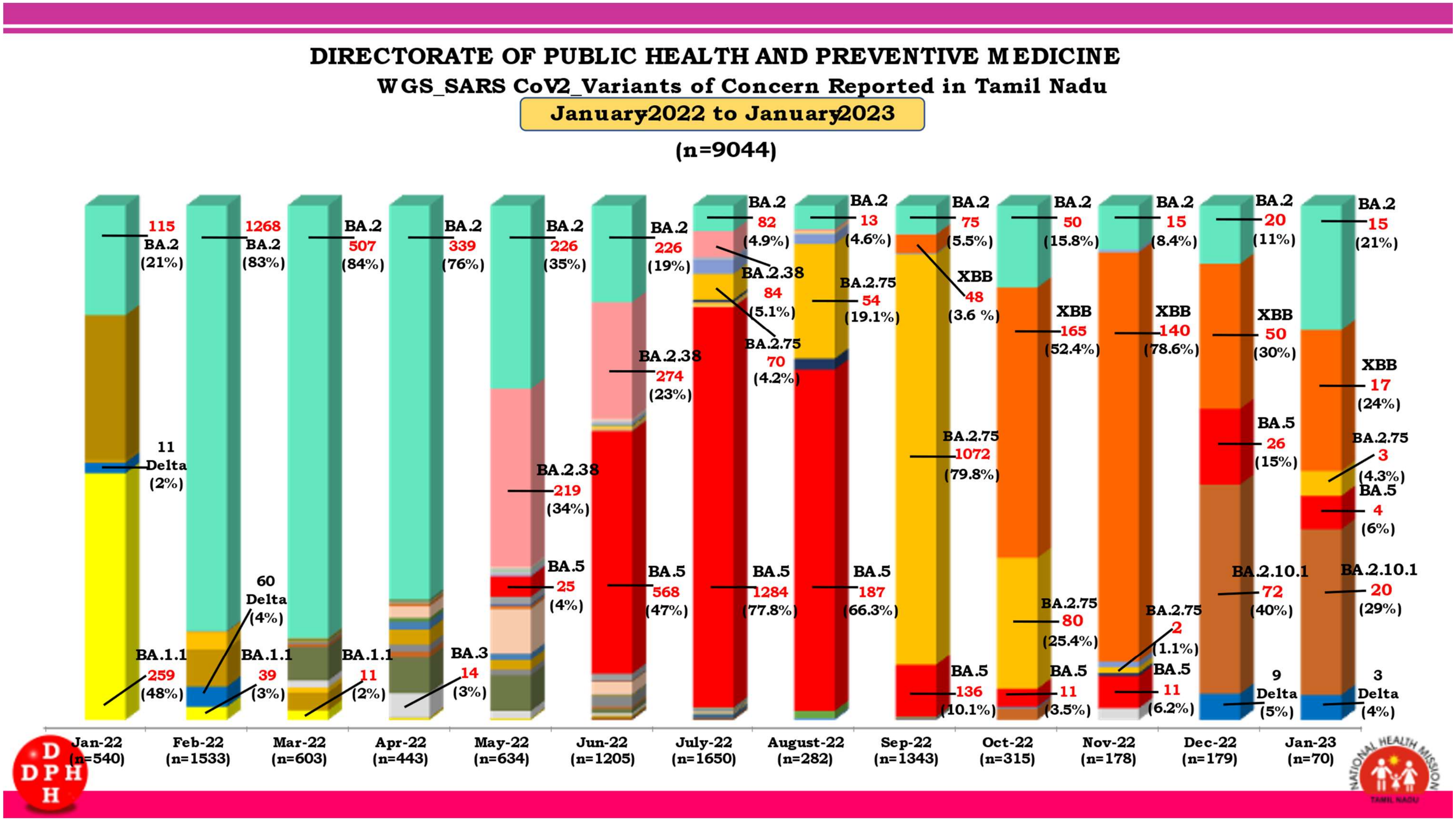

**Supplemental Figure 2:** Threshold of quality control for consensus generation of sequences

| Sequence name | QC | Clade | Pango lineage (Nextclade) | WHO name | Mut. | non-ACGTN | Ns | Cov. | Gaps | Ins. | FS | SC |
|---|---|---|---|---|---|---|---|---|---|---|---|---|
| 2019-nCoV_MN908947\|Sample 228 | N M P C F S | 22F | XBB.1 | Omicron | 91 | 0 | 0 | 99.5% | 57 | 0 | 1 (1) | 0 |
| 2019-nCoV_MN908947\|Sample 27 | N M P C F S | 22F | XBB.5 | Omicron | 104 | 0 | 0 | 99.6% | 63 | 4 | 1 (1) | 0 |
| 2019-nCoV_MN908947\|Sample 178 | N M P C F S | 22F | XBB.1 | Omicron | 100 | 0 | 0 | 99.7% | 84 | 6 | 1 (1) | 0 (1) |
| 2019-nCoV_MN908947\|Sample 206 | N M P C F S | 22F | XBB.5 | Omicron | 88 | 0 | 0 | 99.6% | 88 | 0 | 1 (1) | 0 |
| 2019-nCoV_MN908947\|94 | N M P C F S | 22F | XBB.3 | Omicron | 93 | 0 | 0 | 99.5% | 143 | 0 | 2 (2) | 0 |
| 2019-nCoV_MN908947\|Sample 243 | N M P C F S | 23A | XBB.1.5 | Omicron | 94 | 0 | 0 | 99.6% | 126 | 0 | 2 (2) | 0 (1) |
| 2019-nCoV_MN908947\|Sample 208 | N M P C F S | 23A | XBB.1.5 | Omicron | 83 | 0 | 0 | 99.5% | 282 | 1 | 1 (1) | 0 (1) |
| 2019-nCoV_MN908947\|Sample 18 | N M P C F S | 22F | XBB.3 | Omicron | 96 | 0 | 0 | 99.6% | 208 | 4 | 2 (2) | 0 |
| 2019-nCoV_MN908947\|Sample 242 | N M P C F S | 22F | XBB.1 | Omicron | 97 | 0 | 0 | 99.6% | 78 | 4 | 2 (2) | 0 (1) |
| 2019-nCoV_MN908947\|Sample 202 | N M P C F S | 22F | XBB.1.9 | Omicron | 87 | 0 | 0 | 99.5% | 89 | 0 | 1 (1) | 0 (1) |
| 2019-nCoV_MN908947\|177 | N M P C F S | 22F | XBB.1.39 | Omicron | 83 | 0 | 0 | 99.5% | 90 | 0 | 1 (1) | 0 (1) |
| 2019-nCoV_MN908947\|Sample 200 | N M P C F S | 22F | XBB.2.1 | Omicron | 94 | 0 | 0 | 99.5% | 176 | 0 | 2 (3) | 0 |
| 2019-nCoV_MN908947\|Sample 234 | N M P C F S | 22F | FL.2 | Omicron | 90 | 0 | 0 | 99.5% | 107 | 0 | 1 (1) | 0 (1) |
| 2019-nCoV_MN908947\|59 | N M P C F S | 22F | XBB | Omicron | 87 | 0 | 0 | 99.6% | 223 | 0 | 3 (4) | 0 |
| 2019-nCoV_MN908947\|Sample 44 | N M P C F S | 22F | XBB.3 | Omicron | 91 | 0 | 0 | 99.5% | 98 | 0 | 3 (3) | 0 |
| 2019-nCoV_MN908947\|Sample 42 | N M P C F S | 22F | XBB | Omicron | 90 | 0 | 0 | 99.5% | 126 | 2 | 3 (4) | 0 |
| 2019-nCoV_MN908947\|Sample 240 | N M P C F S | 22F | XBB.1 | Omicron | 83 | 0 | 0 | 99.5% | 272 | 1 | 3 (3) | 0 |
| 2019-nCoV_MN908947\|Sample 188 | N M P C F S | 22F | XBB.1 | Omicron | 95 | 0 | 0 | 99.6% | 136 | 2 | 3 (4) | 0 (1) |
| 2019-nCoV_MN908947\|Sample 241 | N M P C F S | 22F | FL.5 | Omicron | 89 | 0 | 0 | 99.5% | 93 | 0 | 1 (1) | 0 (1) |
| 2019-nCoV_MN908947\|Sample 180 | N M P C F S | 22F | XBB.1.9 | Omicron | 104 | 0 | 0 | 99.3% | 319 | 8 | 4 (5) | 0 (1) |
| 2019-nCoV_MN908947\|Sample 192 | N M P C F S | 22F | XBB.1 | Omicron | 160 | 0 | 0 | 99.3% | 122 | 10 | 3 (3) | 0 (1) |
| 2019-nCoV_MN908947\|Sample 182 | N M P C F S | 22F | XBB.6 | Omicron | 105 | 0 | 0 | 99.5% | 705 | 0 | 5 (5) | 0 |

| Sequence name | QC | Clade | Pango lineage (Nextclade) | WHO name | Mut. | non-ACGTN | Ns | Cov. | Gaps | Ins. | FS | SC |
|---|---|---|---|---|---|---|---|---|---|---|---|---|
| 2019-nCoV_MN908947\|Sample 199 | N M P C F S | 22F | XBB.1 | Omicron | 84 | 0 | 0 | 99.6% | 284 | 0 | 1 (1) | 0 |
| 2019-nCoV_MN908947\|Sample 209 | N M P C F S | 23A | XBB.1.5 | Omicron | 91 | 0 | 0 | 99.5% | 57 | 0 | 1 (1) | 0 (1) |
| 2019-nCoV_MN908947\|Sample 217 | N M P C F S | 22F | XBB.3 | Omicron | 94 | 0 | 0 | 99.6% | 78 | 0 | 1 (1) | 0 |
| 2019-nCoV_MN908947\|Sample 238 | N M P C F S | 23A | XBB.1.5.18 | Omicron | 92 | 0 | 0 | 99.6% | 79 | 0 | 1 (1) | 0 (1) |
| 2019-nCoV_MN908947\|Sample 191 | N M P C F S | 22F | XBB.1 | Omicron | 101 | 0 | 0 | 99.6% | 56 | 0 | 0 | 0 |
| 2019-nCoV_MN908947\|163 | N M P C F S | 22F | XBB | Omicron | 89 | 0 | 0 | 99.6% | 57 | 0 | 1 (1) | 0 |
| 2019-nCoV_MN908947\|Sample 195 | N M P C F S | 22F | XBB.1 | Omicron | 94 | 0 | 0 | 99.5% | 91 | 0 | 1 (1) | 0 |
| 2019-nCoV_MN908947\|Sample 143 | N M P C F S | 22F | XBB.3 | Omicron | 90 | 0 | 0 | 99.6% | 57 | 0 | 1 (1) | 0 |
| 2019-nCoV_MN908947\|Sample 181 | N M P C F S | 22F | XBB.3 | Omicron | 91 | 0 | 0 | 99.5% | 57 | 0 | 1 (1) | 0 |
| 2019-nCoV_MN908947\|Sample 197 | N M P C F S | 22F | XBB.3 | Omicron | 98 | 0 | 0 | 99.6% | 78 | 0 | 1 (1) | 0 |
| 2019-nCoV_MN908947\|161 | N M P C F S | 22F | XBB.3 | Omicron | 92 | 0 | 0 | 99.6% | 57 | 0 | 1 (1) | 0 |
| 2019-nCoV_MN908947\|Sample 244 | N M P C F S | 22F | FL.5 | Omicron | 97 | 0 | 0 | 99.5% | 153 | 0 | 1 (1) | 0 (1) |
| 2019-nCoV_MN908947\|Sample 215 | N M P C F S | 22F | XBB.1 | Omicron | 83 | 0 | 0 | 99.5% | 175 | 0 | 1 (1) | 0 (1) |
| 2019-nCoV_MN908947\|Sample 229 | N M P C F S | 22F | XBB.1 | Omicron | 95 | 0 | 0 | 99.7% | 57 | 0 | 1 (1) | 0 (1) |
| 2019-nCoV_MN908947\|Sample 190 | N M P C F S | 22F | XBB.2.8 | Omicron | 94 | 0 | 0 | 99.5% | 57 | 1 | 1 (1) | 0 |
| 2019-nCoV_MN908947\|Sample 205 | N M P C F S | 22F | XBB.5 | Omicron | 95 | 0 | 0 | 99.6% | 57 | 0 | 1 (1) | 0 |
| 2019-nCoV_MN908947\|Sample 233 | N M P C F S | 22F | XBB.2 | Omicron | 96 | 0 | 0 | 99.5% | 57 | 0 | 1 (1) | 0 |
| 2019-nCoV_MN908947\|Sample 203 | N M P C F S | 22F | XBB | Omicron | 96 | 0 | 0 | 99.7% | 87 | 0 | 1 (1) | 0 |
| 2019-nCoV_MN908947\|Sample 214 | N M P C F S | 22F | XBB | Omicron | 96 | 0 | 0 | 99.7% | 87 | 0 | 1 (1) | 0 |
| 2019-nCoV_MN908947\|Sample 226 | N M P C F S | 22F | XBB.1 | Omicron | 94 | 0 | 0 | 99.5% | 82 | 0 | 1 (1) | 0 (1) |
| 2019-nCoV_MN908947\|Sample 230 | N M P C F S | 22F | XBB | Omicron | 97 | 0 | 0 | 99.5% | 166 | 0 | 1 (1) | 0 |
| 2019-nCoV_MN908947\|Sample 211 | N M P C F S | 23A | XBB.1.5 | Omicron | 95 | 0 | 0 | 99.5% | 71 | 0 | 0 | 0 (1) |

| Sequence name | QC | Clade | Pango lineage (Nextclade) | WHO name | Mut. | non-ACGTN | Ns | Cov. | Gaps | Ins. | FS | SC |
|---|---|---|---|---|---|---|---|---|---|---|---|---|
| 2019-nCoV_MN908947\|Sample 189 | N M P C F S | 22F | XBB.1.9 | Omicron | 89 | 0 | 0 | 99.6% | 56 | 0 | 0 | 0 (1) |
| 2019-nCoV_MN908947\|Sample 194 | N M P C F S | 22F | XBB.1.15 | Omicron | 93 | 0 | 0 | 99.6% | 77 | 0 | 0 | 0 (1) |
| 2019-nCoV_MN908947\|Sample 225 | N M P C F S | 22F | XBB.1 | Omicron | 94 | 0 | 0 | 99.5% | 56 | 0 | 0 | 0 (1) |
| 2019-nCoV_MN908947\|Sample 198 | N M P C F S | 22F | XBB.3 | Omicron | 87 | 0 | 0 | 99.5% | 56 | 0 | 0 | 0 |
| 2019-nCoV_MN908947\|Sample 235 | N M P C F S | 22F | XBB.1 | Omicron | 93 | 0 | 0 | 99.8% | 56 | 0 | 0 | 0 (1) |
| 2019-nCoV_MN908947\|Sample 239 | N M P C F S | 22F | XBB.1 | Omicron | 95 | 0 | 0 | 99.6% | 56 | 0 | 0 | 0 (1) |
| 2019-nCoV_MN908947\|Sample 193 | N M P C F S | 22F | XBB.2 | Omicron | 95 | 0 | 0 | 99.5% | 56 | 0 | 0 | 0 |
| 2019-nCoV_MN908947\|19 | N M P C F S | 22F | XBB.3 | Omicron | 90 | 0 | 0 | 99.5% | 56 | 0 | 0 | 0 |
| 2019-nCoV_MN908947\|144 | N M P C F S | 22F | XBB.1 | Omicron | 88 | 0 | 0 | 99.6% | 57 | 1 | 1 (1) | 0 |
| 2019-nCoV_MN908947\|41 | N M P C F S | 22F | XBB.3 | Omicron | 88 | 0 | 0 | 99.6% | 74 | 0 | 1 (1) | 0 |
| 2019-nCoV_MN908947\|61 | N M P C F S | 22F | XBB.5 | Omicron | 90 | 0 | 0 | 99.5% | 72 | 0 | 1 (1) | 0 |
| 2019-nCoV_MN908947\|Sample 222 | N M P C F S | 22F | XBB.1 | Omicron | 91 | 0 | 0 | 99.5% | 99 | 0 | 1 (1) | 0 (1) |
| 2019-nCoV_MN908947\|Sample 29 | N M P C F S | 22F | XBB.3 | Omicron | 92 | 0 | 0 | 99.5% | 88 | 0 | 1 (1) | 0 |
| 2019-nCoV_MN908947\|Sample 82 | N M P C F S | 22F | XBB.3 | Omicron | 89 | 0 | 0 | 99.5% | 143 | 0 | 1 (1) | 0 |
| 2019-nCoV_MN908947\|95 | N M P C F S | 22F | XBB.3 | Omicron | 93 | 0 | 0 | 99.3% | 126 | 0 | 1 (1) | 0 |
| 2019-nCoV_MN908947\|Sample 124 | N M P C F S | 22F | XBB | Omicron | 91 | 0 | 0 | 99.5% | 235 | 0 | 1 (1) | 0 |
| 2019-nCoV_MN908947\|Sample 213 | N M P C F S | 23A | XBB.1.5 | Omicron | 91 | 0 | 0 | 99.5% | 68 | 1 | 1 (1) | 0 (1) |
| 2019-nCoV_MN908947\|Sample 212 | N M P C F S | 22F | XBB.1 | Omicron | 94 | 0 | 0 | 99.5% | 183 | 0 | 1 (1) | 0 (1) |
| 2019-nCoV_MN908947\|Sample 231 | N M P C F S | 22F | FL.10 | Omicron | 96 | 0 | 0 | 99.5% | 57 | 0 | 1 (1) | 0 (1) |
| 2019-nCoV_MN908947\|Sample 201 | N M P C F S | 22F | XBB.3 | Omicron | 93 | 0 | 0 | 99.6% | 57 | 0 | 1 (1) | 0 |
| 2019-nCoV_MN908947\|Sample 207 | N M P C F S | 22F | XBB.3 | Omicron | 91 | 0 | 0 | 99.5% | 68 | 0 | 1 (2) | 0 |
| 2019-nCoV_MN908947\|Sample 43 | N M P C F S | 22F | XBB.3 | Omicron | 92 | 0 | 0 | 99.5% | 57 | 0 | 1 (1) | 0 |
| 2019-nCoV_MN908947\|133 | N M P C F S | 22F | XBB.3 | Omicron | 90 | 0 | 0 | 99.5% | 56 | 0 | 0 | 0 |
| 2019-nCoV_MN908947\|24 | N M P C F S | 22F | XBB.3 | Omicron | 91 | 0 | 0 | 99.5% | 56 | 0 | 0 | 0 |
| 2019-nCoV_MN908947\|40 | N M P C F S | 22F | XBB.3 | Omicron | 89 | 0 | 0 | 99.5% | 74 | 0 | 0 | 0 |
| 2019-nCoV_MN908947\|77 | N M P C F S | 22F | XBB.5 | Omicron | 91 | 0 | 0 | 99.5% | 56 | 0 | 0 | 0 |
| 2019-nCoV_MN908947\|Sample 232 | N M P C F S | 22F | XBB.2.3 | Omicron | 94 | 0 | 0 | 99.6% | 56 | 0 | 0 | 0 |
| 2019-nCoV_MN908947\|162 | N M P C F S | 22F | XBB.3 | Omicron | 91 | 0 | 0 | 99.5% | 56 | 0 | 0 | 0 |
| 2019-nCoV_MN908947\|60 | N M P C F S | 22F | XBB.3 | Omicron | 90 | 0 | 0 | 99.6% | 61 | 0 | 0 | 0 |
| 2019-nCoV_MN908947\|Sample 227 | N M P C F S | 22F | XBB.1 | Omicron | 91 | 0 | 0 | 99.5% | 128 | 0 | 0 | 0 (1) |
| 2019-nCoV_MN908947\|174 | N M P C F S | 22F | XBB.3 | Omicron | 93 | 0 | 0 | 99.6% | 56 | 0 | 0 | 0 |
| 2019-nCoV_MN908947\|Sample 160 | N M P C F S | 22F | XBB.3 | Omicron | 91 | 0 | 0 | 99.5% | 89 | 0 | 0 | 0 |
| 2019-nCoV_MN908947\|Sample 184 | N M P C F S | 22F | XBB.1.9 | Omicron | 90 | 0 | 0 | 99.5% | 56 | 0 | 0 | 0 (1) |
| 2019-nCoV_MN908947\|Sample 221 | N M P C F S | 22F | XBB.2 | Omicron | 93 | 0 | 0 | 99.7% | 56 | 0 | 0 | 0 |
| 2019-nCoV_MN908947\|52 | N M P C F S | 22F | XBB.1 | Omicron | 87 | 0 | 0 | 99.5% | 56 | 0 | 0 | 0 |
| 2019-nCoV_MN908947\|Sample 237 | N M P C F S | 23A | XBB.1.5.18 | Omicron | 94 | 0 | 0 | 99.5% | 56 | 0 | 0 | 0 (1) |
| 2019-nCoV_MN908947\|Sample 28 | N M P C F S | 22F | XBB.3 | Omicron | 93 | 0 | 0 | 99.7% | 90 | 0 | 0 | 0 |
| 2019-nCoV_MN908947\|76 | N M P C F S | 22F | XBB.3 | Omicron | 93 | 0 | 0 | 99.5% | 56 | 0 | 0 | 0 |
| 2019-nCoV_MN908947\|Sample 185 | N M P C F S | 22F | XBB.1.9 | Omicron | 91 | 0 | 0 | 99.7% | 86 | 0 | 0 | 0 (1) |
| 2019-nCoV_MN908947\|Sample 186 | N M P C F S | 22F | XBB.1.9 | Omicron | 92 | 0 | 0 | 99.4% | 30 | 0 | 0 | 0 (1) |
| 2019-nCoV_MN908947\|Sample 187 | N M P C F S | 22F | XBB.1.9 | Omicron | 92 | 0 | 0 | 99.5% | 53 | 0 | 0 | 0 (1) |
| 2019-nCoV_MN908947\|Sample 204 | N M P C F S | 22F | XBB.3 | Omicron | 89 | 0 | 0 | 99.6% | 56 | 0 | 0 | 0 |
| 2019-nCoV_MN908947\|Sample 179 | N M P C F S | 22F | XBB.1 | Omicron | 90 | 0 | 0 | 99.5% | 56 | 0 | 0 | 0 |
| 2019-nCoV_MN908947\|Sample 223 | N M P C F S | 22F | XBB.2 | Omicron | 94 | 0 | 0 | 99.6% | 127 | 0 | 0 | 0 |

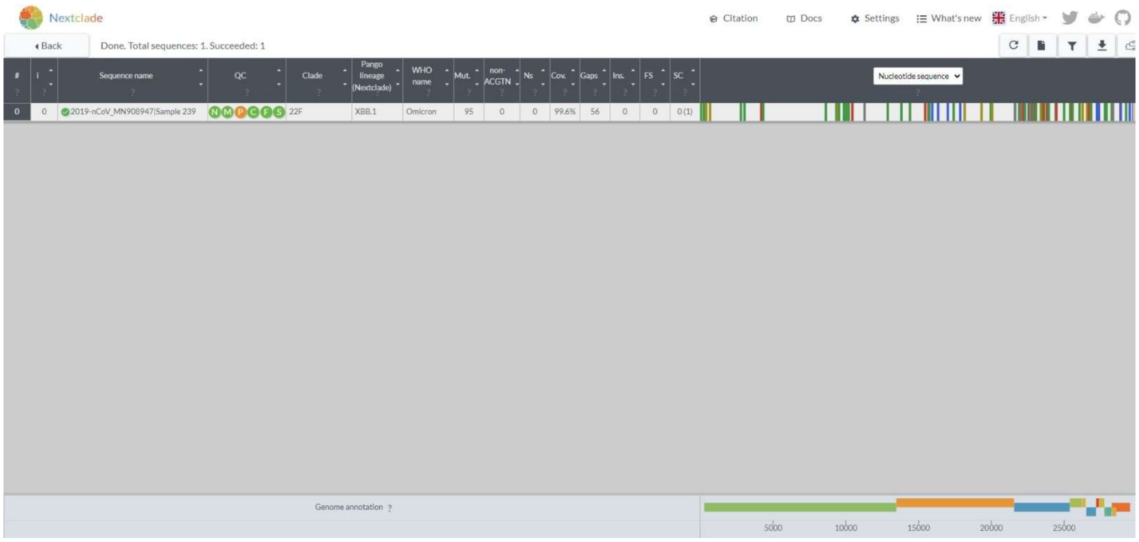
Nextclade
Citation
Docs
Settings
What's new
English
Back
Done. Total sequences: 1. Succeeded: 1
#
i
Sequence name
QC
Clade
Pango lineage (Nextclade)
WHO name
Mut.
non-ACGTN
Ns
Cov.
Gaps
Ins.
FS
SC
Nucleotide sequence
0
0
2019-nCoV_MN908947|Sample 239
N M P C F S
22F
XBB.1
Omicron
95
0
0
99.6%
56
0
0
0 (1)
Genome annotation ?
5000
10000
15000
20000
25000
